# A pillar[5]arene-based [2]rotaxane lights up mitochondria†
†Electronic supplementary information (ESI) available: Experimental details, NMR spectra, and other materials. See DOI: 10.1039/c6sc00036c


**DOI:** 10.1039/c6sc00036c

**Published:** 2016-01-21

**Authors:** Guocan Yu, Dan Wu, Yang Li, Zhihua Zhang, Li Shao, Jiong Zhou, Qinglian Hu, Guping Tang, Feihe Huang

**Affiliations:** a State Key Laboratory of Chemical Engineering , Center for Chemistry of High-Performance & Novel Materials , Department of Chemistry , Zhejiang University , Hangzhou 310027 , P. R. China . Email: fhuang@zju.edu.cn ; Fax: +86-571-8795-3189 ; Tel: +86-571-8795-3189; b Department of Chemistry , Institute of Chemical Biology and Pharmaceutical Chemistry , Zhejiang University , Hangzhou 310027 , P. R. China; c College of Biological and Environmental Engineering , Zhejiang University of Technology , Hangzhou 310014 , P. R. China

## Abstract

Subcellular organelle-specific reagents for simultaneous targeting, imaging and treatment are highly desirable for cancer therapy. However, it remains a challenge to fabricate a single molecular platform containing a targeting group, imaging and therapeutic agents through traditional synthesis. Due to their superior sensitivity and photostability, fluorescent probes with aggregation-induced emission (AIE) characteristics have attracted more and more attention in studying the process of translocation, drug release, and excretion of nanomedicines *in vitro* or *in vivo*. We construct a pillar[5]arene-based [2]rotaxane (**R1**) by employing tetraphenylethene (TPE) and triphenylphosphonium (TPP) moieties as stoppers; the TPE unit retains the aggregation-induced emission (AIE) attribute and the TPP group is used as a mitochondria-targeting agent. **R1** exhibits enhanced AIE, high specificity to mitochondria, and superior photostability. By introducing doxorubicin (DOX) into **R1**, prodrug **R2** is constructed as a dual-fluorescence-quenched Förster resonance energy transfer (FRET) system, in which the TPE-based axle acts as a donor fluorophore and the DOX unit acts as the acceptor. Upon hydrolysis of **R2** in endo/lysosomes, the fluorescences of the carrier and the drug recover. **R1** is further utilized as a drug delivery platform to conjugate other anticancer drugs containing amine groups through imine formation to prepare prodrugs. The anticancer drugs are released from these prodrugs in the cells upon hydrolysis of the pH-responsive imine bonds.

## Abstract 2

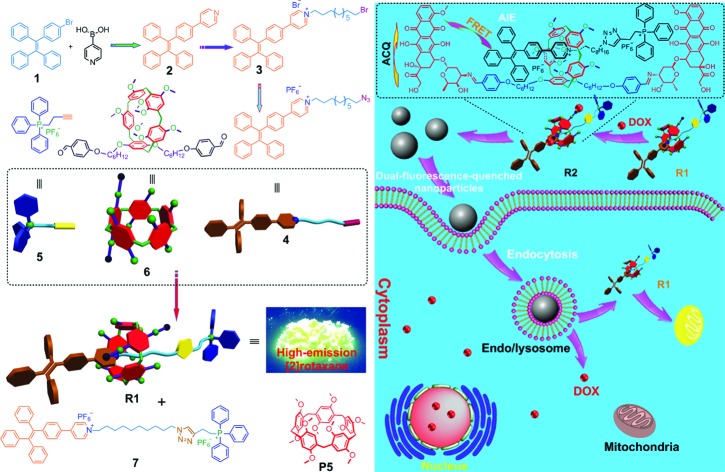
Here we integrate diagnostic and therapeutic agents into a mitochondria-targeting [2]rotaxane, which can be utilized as a drug delivery platform to conjugate anticancer drugs to prepare prodrugs for efficient targeted drug delivery.

## Introduction

Mitochondria, double-membrane-bound subcellular organelles, are central to eukaryotic cells regulating various biochemical processes, such as consuming ∼95% of the O_2_ inspired to generate most of the cell's ATP, generating most of the cellular reactive oxygen species, regulating the cellular redox state, and initiating cellular apoptosis.^1^ Consequently, there exists considerable interest in designing mitochondria-targeting molecules to report on and manipulate mitochondrial function, which is responsible for a wide range of human diseases, including diabetes, cancers and neurodegenerative diseases.^2^ By employing ion channel pumps and oxidation pathways, mitochondria maintain a constant membrane potential of *ca.* –180 mV across their lipid bilayers, approximately twice that of the plasma membrane of excitable cells and six times higher than the plasma membrane of nonexcitable cells, which is unprecedented in any other organelles.^3^ This unique nature of the mitochondrial membrane distinguishes it from its intracellular counterparts and offers a unique chemical opportunity for selectively targeting the mitochondria.^4^ The use of lipophilic cations, for example triphenylphosphonium (TPP), as selective targeting agents has been explored by using the negative potential gradient of the organelle as an electrostatic driving force, which has enabled the delivery of a wide range of molecules to mitochondria without the need to alter gene or protein structures.^5^

Fluorescent probes that can selectively light up cellular mitochondria are powerful tools for monitoring the morphological changes and manipulating mitochondrial functions. Conventional fluorescent dyes, such as 2,1,3-benzothiadiazole, *o*-phenylenediamine, rhodamine dyes, lanthanide complexes and boron-dipyrromethene, have been developed to selectively stain the mitochondria.^6^ Due to the aggregation-caused quenching (ACQ) effect under the driving forces of intermolecular π–π stacking and hydrophobic interactions, these fluorophores are always used in very dilute states in the imaging process and thus can be photobleached quickly when a harsh laser beam is used as the excitation light source. Development of novel fluorophores free of the ACQ effect but with high emission efficiency and excellent photostability in the aggregated state is a challenging task.^7^

In sharp contrast to the ACQ effect, Tang *et al.* developed a novel class of organic luminogens with an extraordinary aggregation-induced emission (AIE) feature, which is exactly opposite to the ACQ systems.^8^ The distinctive organic luminogens, with tetraphenylethene (TPE) and hexaphenylsilole (HPS) being typical examples, are non-emissive in solution but are induced to luminesce intensely in the aggregated state through restriction of intramolecular rotation (RIR) of the benzene rings.^9^ On account of their unique fluorescence turn-on properties with high sensitivity and contrast, a series of AIE-based fluorescent probes have emerged for living cell imaging and the detection of a wide range of biomolecules over the past decade. In order to effectively restrict the intramolecular rotation of the aromatic rings to achieve the AIE effect, various methods including covalent and noncovalent modifications have been employed. In comparison to covalent functionalizations, supramolecular approaches to modify the AIE-active fluorogens are especially important for biocompatibilization and bioapplications, because the unique properties of the fluorogens can be effectively preserved.^10^ More importantly, it remains a challenge to introduce targeting groups, subcellular organelle-specific imaging agents and therapeutic agents into a single molecular platform through traditional synthesis. The importance of mechanically interlocked molecules (MIMs), such as rotaxanes and catenanes, has grown dramatically in recent years because of their contribution in topology and inventive applications in nanoscience.^11^ For example, the use of rotaxane-based architectures for biological applications was reported recently.11f,q It will be interesting to know whether the restriction of the intramolecular rotation of aromatic rings of AIE-active fluorogens can be achieved by forming MIMs, thereby effectively enhancing their AIE effect. More importantly, targeting, diagnostic and therapeutic agents can be introduced into a MIM by conjugating the functional groups on the different components of the mechanically interlocked structure, endowing the MIM with theranostic functions.

**Scheme 1:**
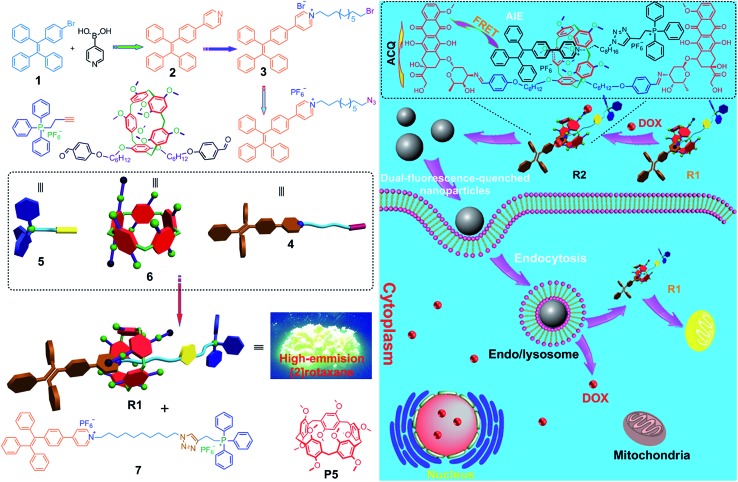
(Left) Synthetic route to **R1** and the corresponding dumbbell-shaped **7** and chemical structures of some compounds used here. (Right) Schematic illustration of the preparation of a mitochondria-targeting probe-inspired prodrug **R2** and possible cellular pathways of the dual-fluorescence-quenched **R2** nanoparticles. The fluorescences of both TPE and DOX in the nanoparticles self-assembled from **R2** are inactivated by the energy transfer relay (ETR) effect, mediated by Förster resonance energy transfer (FRET) and aggregation-caused quenching. The ETR between DOX and **R1** is interrupted inside the endo/lysosomes arising from the hydrolysis of the imine bonds. The hydrolyzed DOX and **R1** escape from endosomes/lysosomes, move through the cytoplasm toward the nucleus and mitochondria, and cross the mitochondrial membrane and the nuclear membrane, respectively, and the “silenced” fluorescence “wakes up”.

Herein, we fabricated a pillar[5]arene-based [2]rotaxane (**R1**) by using TPE and triphenylphosphonium (TPP) groups as stoppers (Scheme 1); the TPE group acted as an AIE-active fluorogen and TPP was utilized as a mitochondria-targeting unit. Compared with that of the free axle, the AIE effect of the rotaxane was enhanced significantly because the intramolecular rotation of the benzene rings of the TPE unit was restricted upon formation of the [2]rotaxane structure. This rotaxane could light up mitochondria specifically with excellent targeting ability and superior photostability. Furthermore, by introducing doxorubicin (DOX) into **R1**, prodrug **R2** was constructed as a dual-fluorescence-quenched Förster resonance energy transfer (FRET) system, in which the TPE-based axle acted as the donor fluorophore and the DOX unit acted as the acceptor. Upon hydrolysis of **R2** in endo/lysosomes, the fluorescences of the carrier and the drug recovered, displaying a dual-color fluorogenic process. **R1** was further utilized as a drug delivery platform to conjugate other anticancer drugs containing amine groups through imine formation. The anticancer drugs were activated in the cells upon hydrolysis of the pH-responsive imine bonds.

## Results and discussion

The host–guest interactions among **4**, **5** and **6** were investigated by employing 1,4-dimethoxypillar[5]arene (**P5**) as a model host (Scheme 1). By analyzing the ^1^H NMR and electrospray ionization mass spectrometry (ESI-MS) spectra, we knew that **P5** could interact with **4** containing a long alkyl chain and a cationic pyridinium unit driven by the cooperativity of cation···π and multiple C–H···π interactions. While no host–guest complexation between **P5** and **5** was monitored due to the steric hindrance and the shortage of effective driving forces (for details, see ESI, Fig. S31–S39†). The copper(i)-catalyzed azide–alkyne cycloaddition (CuAAC) reaction was used to synthesize **R1**. The alkyne-terminated **5** was added to a mixture of the wheel **6** and the semi-blocked rod-like **4**, together with TBTA and Cu(CH_3_CN)_4_PF_6_. Purification of the crude product from the reaction mixture by silica gel chromatography afforded **R1** in a moderate yield (28%). Various methods, including ^1^H, ^13^C, ^31^P, DEPT135, COSY, NOESY, HMBS and HSQC NMR spectroscopies (Fig. 2 and S18–S30†), were employed to confirm the formation of [2]rotaxane **R1**. The ESI-MS data of **R1** contained a peak at *m*/*z* 1019.5 (Fig. S20†), corresponding to [**R1** – 2PF_6_]^2+^, which provided direct evidence for the formation of **R1**.

**Fig. 1:**
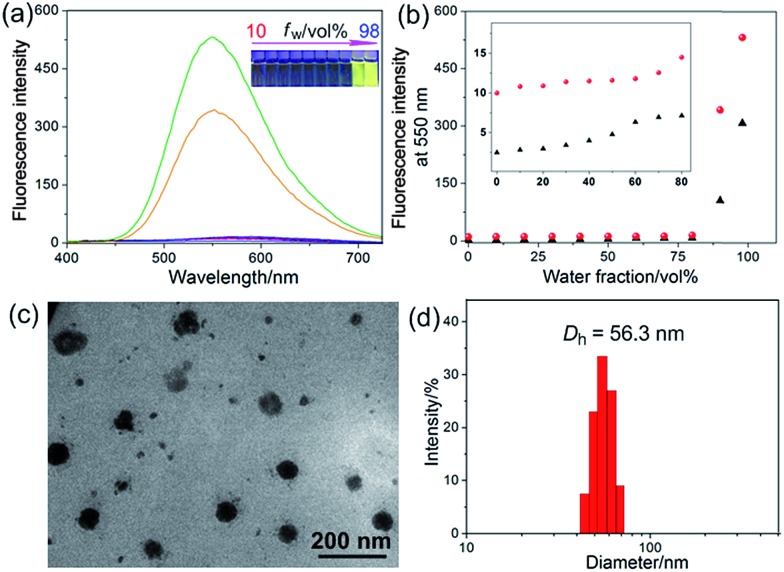
(a) Fluorescence spectra of **R1** in mixtures of THF and water with different *f*_w_ values. Inset: a fluorescent photo of **R1** in mixtures of THF and water with different *f*_w_ values. (b) Plot of the emission intensity at 550 nm *vs. f*_w_ of the aqueous mixtures: (▲) **7**; (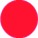) **R1**. The concentrations of **7** and **R1** were 2.00 × 10^–5^ M. (c) TEM image of the aggregates self-assembled from **R1** in water. (d) DLS data of the nanoparticles self-assembled from **R1** in water.

Compared with the free axle **7**, **R1** exhibited an enhancement of the AIE effect upon the rotaxane formation (Fig. 1a and b). When the water fraction (*f*_w_) value reached 98%, the quantum yield (*Φ*_F_) value of **R1** was measured to be 28.6% using fluorescein sodium as the standard (*Φ*_F_ = 0.95 in 0.1 M NaOH aqueous solution), about 2-fold that of **7** under the same conditions (*Φ*_F_ = 13.2%). The intramolecular rotation of the aromatic rings of the TPE group was restricted and the nonradiative decay channels were blocked effectively by forming the rotaxane structure. On the other hand, the hydrophobicity of the AIE-active fluorogen was enhanced significantly by introducing hydrophobic pillar[5]arene **6** into this rotaxane. As a consequence, the aggregation of **R1** in the mixture of THF and H_2_O was greater than that of free **7** as the *f*_w_ value increased, resulting in the enhancement of the AIE effect.

Then a reprecipitation technique was employed to prepare nanoparticles (NPs) that were suitable for cellular uptake by slow addition of 10 μL of a DMSO solution of **R1** into water with stirring. Transmission electron microscopy (TEM) and dynamic light scattering (DLS) were utilized to elucidate the morphology and size of the nanoaggregates. Globular NPs with about 50 nm in diameter were observed in the TEM image (Fig. 1c), in agreement with the result obtained from the DLS experiment (56.3 nm, Fig. 1d). From 3-(4′,5′-dimethyl-2′-thiazolyl)-2,5-diphenyltetrazolium bromide (MTT) assay, minimal influence on relative cell viability towards both HeLa and HEK293 cells incubated with **R1** NPs for 24 h was observed with concentrations ranging from 5 to 25 μM (Fig. S44†), indicating the excellent biocompatibility and low toxicity of this rotaxane.

Next, the internalization pathways of the NPs self-assembled from **R1** were studied using flow cytometry by applying various endocytosis inhibitors.^12^ Uptake of **R1** NPs by HeLa and HEK293 cells was almost completely inhibited at 4 °C (the low temperature is beneficial to minimize the metabolism of cell plasma membrane), demonstrating the energy-dependent nature of particle uptake (Fig. S45†). Moreover, the cellular uptake of **R1** NPs by HeLa and HEK293 cells was effectively blocked by sucrose and amiloride-HCl, suggesting that the internalization of these particles was mainly mediated by macropinocytosis- and clathrin-mediated endocytosis rather than the caveolae-mediated pathway.12*b* These pathways allowed **R1** NPs to undergo the endo/lysosomal transport for intracellular delivery of **R1**.

**Fig. 2:**
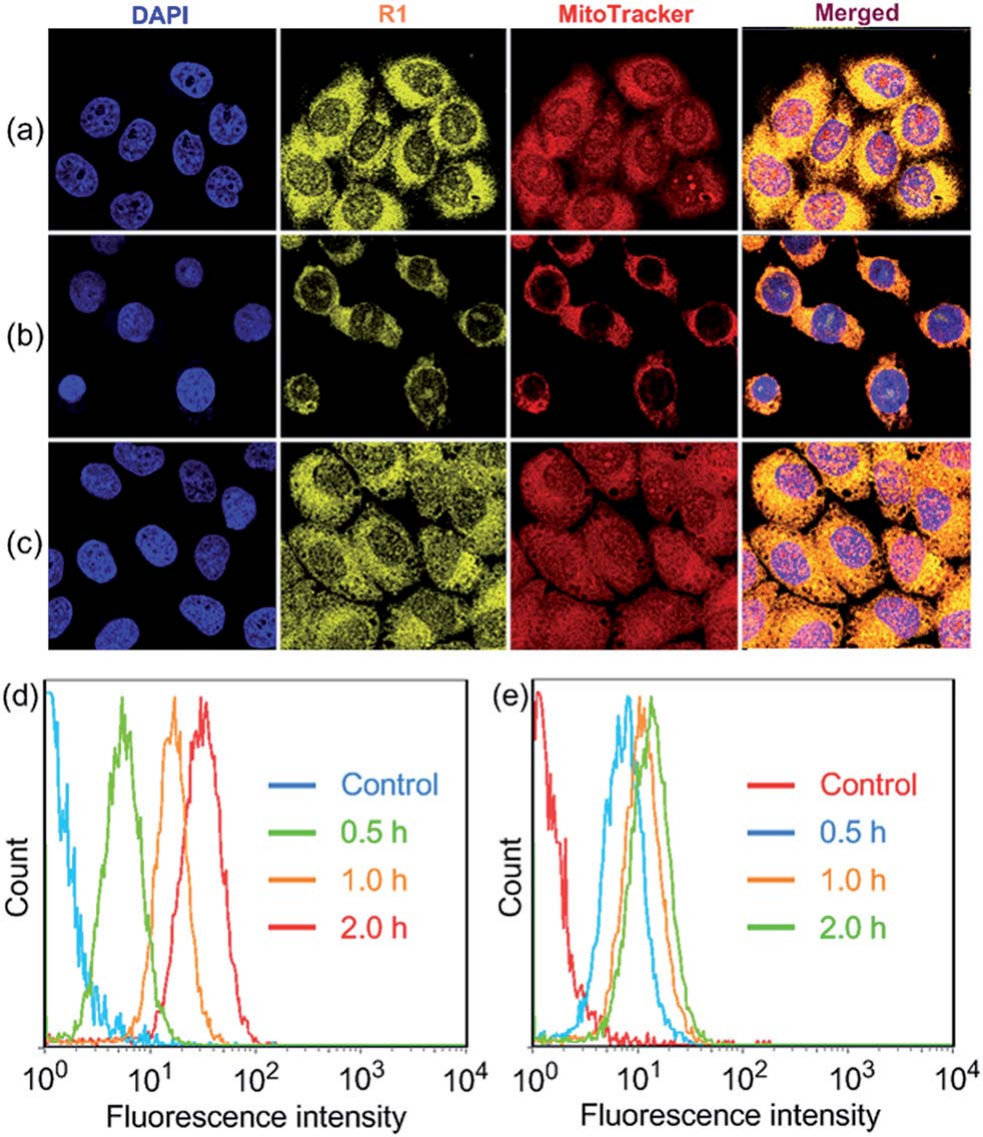
Confocal microscopy images of the cells stained with **R1** (2.00 μM) and MitoTracker Red (100 nM): (a) HeLa cells; (b) HEK293 cells; (c) HeLa cells after incubation with CCCP (10 μM). The quantitative analysis of **R1** fluorescence intensity in (d) HeLa and (e) HEK293 cells by flow cytometry upon incubating the cells with **R1** NPs (2.00 μM) for different time periods.

**R1** was then assessed for its ability to localize and stain mitochondria in HeLa and HEK293 cells by confocal laser scanning microscopy (CLSM). Both cells were cultured with **R1** NPs for 2 h, which were co-stained with MitoTracker Red (a commercial mitochondrial stain). As shown in Fig. 2a, **R1** stained specifically the mitochondrial region in HeLa cells. The yellow fluorescence signal from the rotaxane was well-overlapped with the red fluorescence signal from MitoTracker Red. The reticulum structures of mitochondria were clearly visible with the aid of the yellow fluorescence from **R1**. The Pearson's correlation coefficient, used to quantify the overlap between the **R1** emission and the MitoTracker Red emission, was determined to be 0.94, indicating the specific targeting of **R1** on mitochondria.3*c* For the HEK293 cells (Fig. 2b), the Pearson's correlation coefficient was 0.82, suggesting that the probe exhibited better selectivity for mitochondria in HeLa cells than that in HEK293 cells. The discrepancy in Pearson's correlation coefficients was because carcinoma HeLa cells have a more negative mitochondrial membrane potential than HEK293 cells (approximately 60 mV potential difference between normal cells and carcinoma cells).3*c* Flow cytometry measurements were conducted to investigate the cellular uptake of **R1** by HeLa and HEK293 cells. As shown in Fig. 2d, the uptake of **R1** by HeLa cells was time-dependent and increased rapidly by extending the culture time. From comparison of Fig. 2d and e, HeLa cells had a faster uptake rate and higher intracellular accumulation than HEK293 cells, revealing that the uptake of **R1** by HeLa cells was more efficient. This difference was also due to the higher mitochondrial membrane potential of HeLa cells compared with that of HEK293 cells, which favors TPP-based nanocarrier uptake for carcinoma cells.

Carbonyl cyanide *m*-chlorophenyl hydrazone (CCCP), an uncoupler, can rapidly acidulate the mitochondria and make ATP synthase disordered, which results in the decrease of the mitochondrial membrane potential (Δ*Ψ*_m_).3*b* According to the Nernst equation, the Δ*Ψ*_m_ value decreases around 40 mV when the pH of mitochondria decreases by about 0.7 upon treatment of the cells with 20 μM CCCP.1*a* In order to test the tolerance of **R1** and MitoTracker Red to the change of mitochondrial Δ*Ψ*_m_, HeLa cells were pretreated with CCCP (10 μM) for 30 min before culturing with **R1** and MitoTracker Red, respectively. As shown in Fig. 2c, red fluorescence was observed in the cell nucleus, indicating that the specificity and sensitivity of MitoTracker Red was decreased significantly because of the decrease of Δ*Ψ*_m_ when the cells were pretreated with CCCP.3*b* It should be noted that the specificity and sensitivity of **R1** to mitochondria were perfectly retained in CCCP-pretreated HeLa cells (Fig. 2c), because the lipophilicity of **R1** was much higher than that of MitoTracker Red by introducing the hydrophobic TPE stopper and pillar[5]arene wheel into the [2]Rotaxane, which played an important role in retaining the specificity and sensitivity of **R1** to mitochondria.

Moreover, **R1** displayed excellent photostability. No significant difference was observed between the first scan and the 50th scan in CLSM images, and the signal loss of **R1** was measured to be 10.6% after 50 scans (ESI, Fig. S46†). On the contrary, the fluorescence signal of MitoTracker Red almost disappeared after 20 scans, and the signal loss was measured to be as high as 76.6% after 20 scans, because MitoTracker Red in the mitochondrial matrix is destroyed by the strong excitation light at low working concentration. For **R1**, the nanoaggregates in the mitochondria enhanced its photostability. The outermost layer of the nanoaggregates might be photobleached upon exposure to the excitation light, while the condensed core of the NPs would not be photobleached or photo-oxidized because of lack of oxygen diffusion into the particles.

**Fig. 3:**
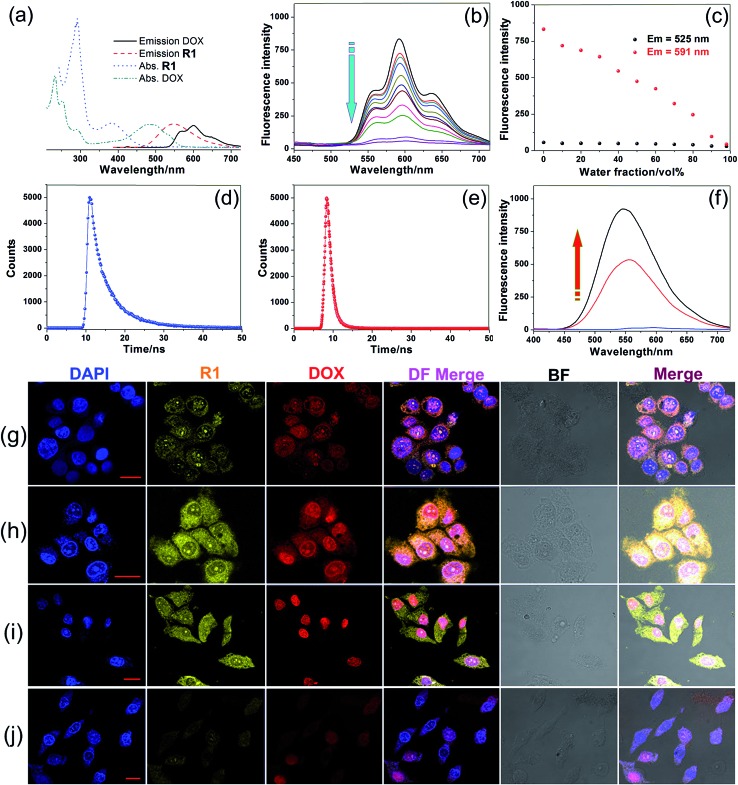
(a) Absorption and fluorescence emission spectra of **R1** and DOX·HCl. (b) Fluorescence spectra of **R2** in THF/water mixtures with different *f*_w_ values. (c) Plot of emission intensities at 525 and 591 nm *vs. f*_w_ of the aqueous mixture. Fluorescence lifetimes of (d) **R1** and (e) **R2**. (f) Recovery of **R1** fluorescence by treating **R2** NPs with normal saline at pH 5.0 for 24 h (the free DOX was removed by dialysis). CLSM images depicting the subcellular distributions of **R1** and DOX in the (g–i) HeLa cells and (j) HEK293 cells. The incubation times were (g) 2 h, (h) 4 h, (i) 8 h and (j) 8 h. Scale bars for all images = 20 μm.

After we knew that **R1** had enhanced AIE, high specificity to mitochondria, and superior photostability, prodrug **R2** was constructed by introducing DOX into **R1**. Fig. 3a shows the absorption and fluorescence emission spectra of **R1** and doxorubicin hydrochloride (DOX·HCl), a typical anticancer drug which can be used for tracing drug delivery and cancer treatment. **R1** is excited by absorbing light with a wavelength of 400 nm, resulting in emission of light in the range of 450–700 nm. We found an overlap between the emission spectrum of **R1** and the absorption spectrum of DOX·HCl, confirming that **R1** could act as a fluorescent donor for the acceptor (DOX·HCl) that absorbs maximally at 500 nm.^13^ Upon the introduction of DOX, the fluorescences of the TPE and DOX chromophores were both inactivated by the energy transfer relay (ETR) effect, mediated by FRET and ACQ. As shown in Fig. 3b, the characteristic emission corresponding to the TPE-based fluorogen was not observed for **R2**, indicating that the AIE behavior disappeared by introducing DOX into this rotaxane, which was ascribed to the emissive energy transfer from the TPE-based fluorogen to DOX, because the distance between the donor and the acceptor was so short that FRET easily took place in **R2**. The efficiency of energy transfer (*Φ*_T_) of this system was calculated to be as high as 81% (ESI, Fig. S47†), indicating that a very efficient energy transfer took place from the TPE-based chromophore to the wheel unit. Moreover, the Förster radius (*R*_0_), the energy transfer rate (*k*_T_) and the donor/acceptor distance were estimated to be 1.99 nm, 0.77, and 1.56 nm, respectively (Fig. 3d and e and ESI, Fig. S47†). The ACQ effect of DOX in the aggregated state reduced the fluorescence intensity by “π–π stacking” of their rigid planar aromatic rings. To verify the ACQ behavior, a solvent dependent aggregation method was employed. The DOX chromophore in **R2** exhibited strong fluorescence at 591 nm when **R2** was well dissolved in THF. However, the fluorescence intensity dramatically decreased upon addition of water (Fig. 3b and c). When the water fraction reached 98 vol%, the fluorescence intensity of the DOX chromophore was nearly 19.3-fold weaker than that in pure THF (Fig. 3c), confirming that the ACQ effect of the DOX chromophore gradually increased along with the aggregation of **R2**.13*h*

The intracellular microenvironment of tumor cells is typically characterized by slightly acid pH in the endosomal (5.0–6.0) and lysosomal (4.0–5.0) compartments. The ETR between TPE and DOX groups could be interrupted inside the endo/lysosomes by hydrolysis of the pH-responsive imine bonds (Fig. 3f),13*d* which was confirmed by ^1^H NMR and the release behavior of DOX at different pH values (ESI, Fig. S48, S51 and S52†). CLSM was also employed to monitor the intracellular pH-triggered hydrolysis of **R2** NPs. HeLa cells were cultured with **R2** NPs for 2, 4 and 8 h, respectively, before imaging. The fluorescence intensity corresponding to DOX and **R1** increased gradually as the incubation time elapsed (Fig. 3g–i), indicating that DOX and **R1** were successfully released from the **R2** NPs in a time-dependent manner. In the “red” fluorescence channel, DOX was distributed in both the cytoplasm and nucleus after culturing the cells with **R2** NPs for 2 h. After 4 h incubation, the fluorescence of DOX was mostly co-localized in the nucleus. For an 8 h incubation, the subcellular distributions of DOX remained nearly unchanged compared with the fluorescence distribution at 4 h, but the fluorescence became brighter. At the same time, no signal corresponding to **R1** was observed in the nucleus; the “yellow” fluorescence of **R1** was localized in the mitochondria throughout this process, suggesting that **R1** maintained its high specificity to mitochondria.

Interestingly, both the fluorescences related to DOX and **R1** were much weaker in HEK293 cells than those in HeLa cells by culturing the cells with **R2** NPs for 8 h under the same conditions (Fig. 3j). The fluorescence intensity of DOX and **R1** in HEK293 cells treated with **R2** NPs for 8 h was even lower than that in HeLa cells treated with **R2** NPs for 2 h. This resulted from the higher cellular uptake of **R2** NPs by HeLa cells compared with HEK293 cells. We hypothesized that **R2** NPs were generally internalized by endocytosis, and were translocated into endo/lysosomes. On account of the acidic pH inside the endo/lysosomes, the ETR effect between DOX and **R1** was interrupted arising by hydrolysis of imine bonds, and the “silenced” fluorescence “woke up”. The hydrolyzed DOX and **R1** escaped from endo/lysosomes, moved through the cytoplasm toward the nucleus and mitochondria, and crossed the mitochondrial membrane and the nuclear membrane, respectively (Scheme 1).

Furthermore, **R1** was also utilized as a drug delivery platform to conjugate other anticancer drugs (gemcitabine, temozolomide and cytarabine hydrochloride) to prepare prodrugs **RGe**, **RTe** and **RCy** through the reaction between the amine groups on the anticancer drugs and the aldehyde units on the pillar[5]arene-based wheel (Fig. S53†). NPs with diameters ranging from 30 to 80 nm were obtained through reprecipitation (ESI, Fig. S49 and S54†). MTT assays were utilized to verify the efficacy of these prodrugs, and the free anticancer drugs were used as control groups. The relative cell viability of the cells incubated with these NPs decreased gradually when the concentration of these prodrugs increased from 5 to 25 μM (ESI, Fig. S57–S60†), demonstrating that **R1** could be used as a nanoprodrug platform to conjugate anticancer drugs containing amine groups. All the DDSs were retained in endo/lysosomes and released the drugs, which entered the active sites and **R1** carriers moved through the cytoplasm toward the mitochondria. Compared with the free anticancer drugs, we could draw a conclusion that the anticancer activity of these drugs was retained. It should be emphasized that the cytotoxicity of **R2** towards HeLa cells was higher than that towards HEK293 cells, indicating that the prodrug selectively accumulated in cancer cells owing to their more negative membrane potential *vs.* normal cells (ESI, Fig. S57†). These results demonstrated that targeting, imaging and therapeutic agents can be introduced into a rotaxane without changing their functions, which is of enormous interest in cancer therapy.

## Conclusions

In summary, we synthesized a pillar[5]arene-based [2]rotaxane by using TPE and TPP groups as stoppers, in which the TPE group acted as a AIE-active fluorogen and TPP acted as a mitochondria-targeting unit. By taking advantage of the mechanically interlocked structure, targeting, imaging and therapeutic agents were integrated into a single molecular platform. On account of its sophisticated topology structure, **R1** exhibited enhanced AIE, high specificity to mitochondria and superior photostability. **R1** was further utilized as a drug delivery platform to conjugate anticancer drugs containing amine groups through imine bonds. The efficacies of the drugs were retained effectively and the resulting nanoprodrugs selectively accumulated in cancer cells owing to their more negative mitochondrial membrane potential than normal cells. Given that the use of a single mechanically interlocked structure containing targeting, imaging and therapeutic agents is unique, we trust this work will inspire the further application of mechanically interlocked molecules in drug delivery, showing their unsurpassable advantages in topological structures.

## Supplementary Material

Supplementary information
