# Supplementary material for: A pillar[5]arene-based [2]rotaxane lights up mitochondria
Source: Chem Sci. 2016 Jan 21;7(5):3017–24. doi: 10.1039/c6sc00036c (PMC6003608; doi:10.1039/c6sc00036c)
Supplement: Supplementary file 1 [file SC-007-C6SC00036C-s001.pdf]

Electronic Supplementary Material (ESI) for Chemical Science.
This journal is © The Royal Society of Chemistry 2016

A pillar[5]arene-based [2]rotaxane lights up mitochondria

Guocan Yu,[a] Dan Wu,[b] Yang Li,[b] Zhihua Zhang,[a] Li Shao,[a] Jiong Zhou,[a] Qinglian Hu,[c] Guping Tang,[b] and Feihe Huang*,[a]

[a] *Department of Chemistry, Center for Chemistry of High-Performance & Novel Materials, Zhejiang University, Hangzhou 310027, P. R. China; Email: fhuang@zju.edu.cn*

[b] *Department of Chemistry, Institute of Chemical Biology and Pharmaceutical Chemistry, Zhejiang University, Hangzhou 310027, P. R. China.*

[c] *College of Biological and Environmental Engineering, Zhejiang University of Technology, Hangzhou 310014, P. R. China.*

Electronic Supplementary Information (50 pages)

1. *Materials and methods*
2. *Synthesis of **R1***
3. *Host–guest complexations between **4**, **5**, and **P5***
4. *Solvent-dependent molecular motion in **R1***
5. *AIE property of 7*
6. *Cytotoxicity evaluation of **R1** towards HeLa and HEK293 cells*
7. *Photostability investigation of **R1** and MitoTracker Red in HeLa cells*
8. *Förster resonance energy transfer in **R2***
9. *Fabrication of nanoprodrugs*

1. Materials and methods

1,10-Dibromodecane, 4-pyridineboronic acid, 4-hydroxybenzaldehyde tris[(1-benzyl-1H-1,2,3-triazol-4-yl)methyl]amine (TBTA), $Cu(CH_3CN)_4PF_6$ and other reagents were commercially available and used as received. Solvents were either employed as purchased or dried according to procedures described in the literature. Compounds **1**[S1] and **6a**[S2] were synthesized according to literature procedures. NMR spectra were recorded on a Bruker Avance DMX 500 spectrophotometer or a Bruker Avance DMX 400 spectrophotometer using the deuterated solvent as the lock and the residual solvent and TMS as the internal reference. Mass spectra were obtained on a Bruker Esquire 3000 plus mass spectrometer (Bruker-Franzen Analytik GmbH Bremen, Germany) equipped with an ESI interface and an ion trap analyzer. HRMS were obtained on a WATERS GCT Premier mass spectrometer. Transmission electron microscopy (TEM) investigations were carried out on a HT-7700 instrument. Fluorescence lifetime measurements were carried on a transient fluorescence spectrometer (Edinburgh Instruments FLS920), and the samples were excited by a 405 nm picosecond pulsed laser at a repetition frequency of 2 MHz. UV-vis spectra were taken on a Shimadzu UV-2550 UV-vis spectrophotometer. The fluorescence experiments were conducted on a RF-5301 spectrofluorophotometer (Shimadzu Corporation, Japan).

Fabrications of Nanoprodrugs. R1 (1.00 mmol) and 2 equiv. of anticancer drug (DOX·HCl, gemcitabine, temozolomide, or cytarabine hydrochloride) were dissolved in anhydrous ethanol (10 mL) in the presence of triethylamine (two drops). The mixture was heated under nitrogen at reflux for 48 h. The organic solvent was evaporated under vacuum, and the residue was dissolved in DMSO. A dimethyl sulfoxide solution (10 μL) of the prodrug was injected into water with controlled stirring to afford the corresponding nanoprodrugs.

Transmission Electron Microscopy (TEM) and Dynamic Light Scattering (DLS) Studies. The nanostructures of **R1** (or the nanoprodrugs) were revealed using TEM. TEM samples were prepared by drop-coating the solution onto a carbon-coated copper grid. TEM experiments were performed on an HT-7700 instrument. The corresponding

solution was left to stand overnight and the insoluble precipitate was eliminated by using a microporous membrane before DLS tests. Dynamic light scattering (DLS) measurements were carried out using a 200 mW polarized laser source Nd:YAG (λ = 532 nm). The polarized scattered light was collected at 90° in a self-beating mode with a Hamamatsu R942/02 photomultiplier. The signals were sent to a Malvern 4700 submicrometer particle analyzer system.

Cell Culture. HeLa and HEK293 cells were cultured in Dulbecco's modified Eagle's medium (DMEM) containing 10% fetal bovine serum (FBS) and 1% penicillin/streptomycin. Cells grew as a monolayer and were detached upon confluence using trypsin (0.5% *w*/*v* in PBS). The cells were harvested from cell culture medium by incubating in trypsin solution for 5 min. The cells were centrifuged, and the supernatant was discarded. A 3 mL portion of serum-supplemented DMEM was added to neutralize any residual trypsin. The cells were resuspended in serum-supplemented DMEM at a concentration of 1×10^4 cells/mL. Cells were cultured at 37 °C and 5% CO_2.

Evaluation of Cytotoxicity. The cytotoxicity of **R1**, **R2**, and other nanoprodrugs against HeLa and HEK293 cells was determined by 3-(4,5-dimethylthiazol-2-yl)-2,5-diphenyl tetrazolium bromide (MTT) assays in a 96-well cell culture plate. All solutions were sterilized by filtration with a 0.22 μm filter before tests. HeLa and HEK293 cells were seeded at a density of 1×10^4 cells/well in a 96-well plate, and incubated for 24 h for attachment. Cells were then incubated with **R1**, **R2**, or the other nanoprodrugs at various concentrations for 24 h. After washing the cells with PBS buffer, 20 μL of a MTT solution (5 mg/mL) was added to each well. After 4 h of incubation at 37 °C, the MTT solution was removed, and the insoluble formazan crystals that formed were dissolved in 100 μL of dimethylsulfoxide (DMSO). The absorbance of the formazan product was measured at 570 nm using a spectrophotometer (Bio-Rad Model 680). Untreated cells in media were used as a control. All experiments were carried out with five replicates.

Cellular uptake assay. HeLa and HEK293 cells at a density of 2×10^5 cells per well were seeded onto 6-well plates and grown for 24 h. The cells without treatment were used

as control. To study the time-dependent cellular uptake, **R1** NPs in fresh DMEM medium was incubated with cells for 0.5, 1, and 2 h. Then the cells were washed with PBS, trypsinized, and re-suspended in PBS. Flow cytometry measurements were conducted using BD FACSEALIBUR with excitation at 405 nm. The mean fluorescence was determined by counting 10,000 events.

For confocal imaging, the tested cells were cultured in the chambers at a density of 5 × 10^5 per mL for 24 h. The cells were incubated with **R1** NPs at 37 °C for 2 h, followed by staining with MitoTracker Red for 30 min. Then the cells were washed with PBS and imaged immediately by confocal laser scanning microscope (CLSM, ZEISS LSM780).

Cell Imaging with Carbonyl Cyanide *m*-Chlorophenylhydrazone (CCCP) Treatment. Cells were grown overnight on a 35 mm petri dish with a cover slip. The cells were incubation with 10 μM CCCP for 30 min. The CCCP treated cells were then stained by **R1** for 2 h and MitoTracker Red for 30 min.

Photostability studies of R1 and MT. Continuous scanning by confocal microscope was used to quantitatively investigate the photostability of **R1** and MitoTracker Red. Two dishes of HeLa cells subcultured from the same source were stained with 2 μM **R1** and 100 nM MitoTracker Red, respectively. With the help of a power meter, excitation power from 405 and 560 nm channels of the microscope were unified (65 μW) and used to irradiate the **R1** and MitoTracker Red stained cells. The initial intensity referred to the first scan of **R1** and MitoTracker Red stained cells was normalized, and the percentage of fluorescence signal loss was calculated.

2. Synthesis of ***R1***

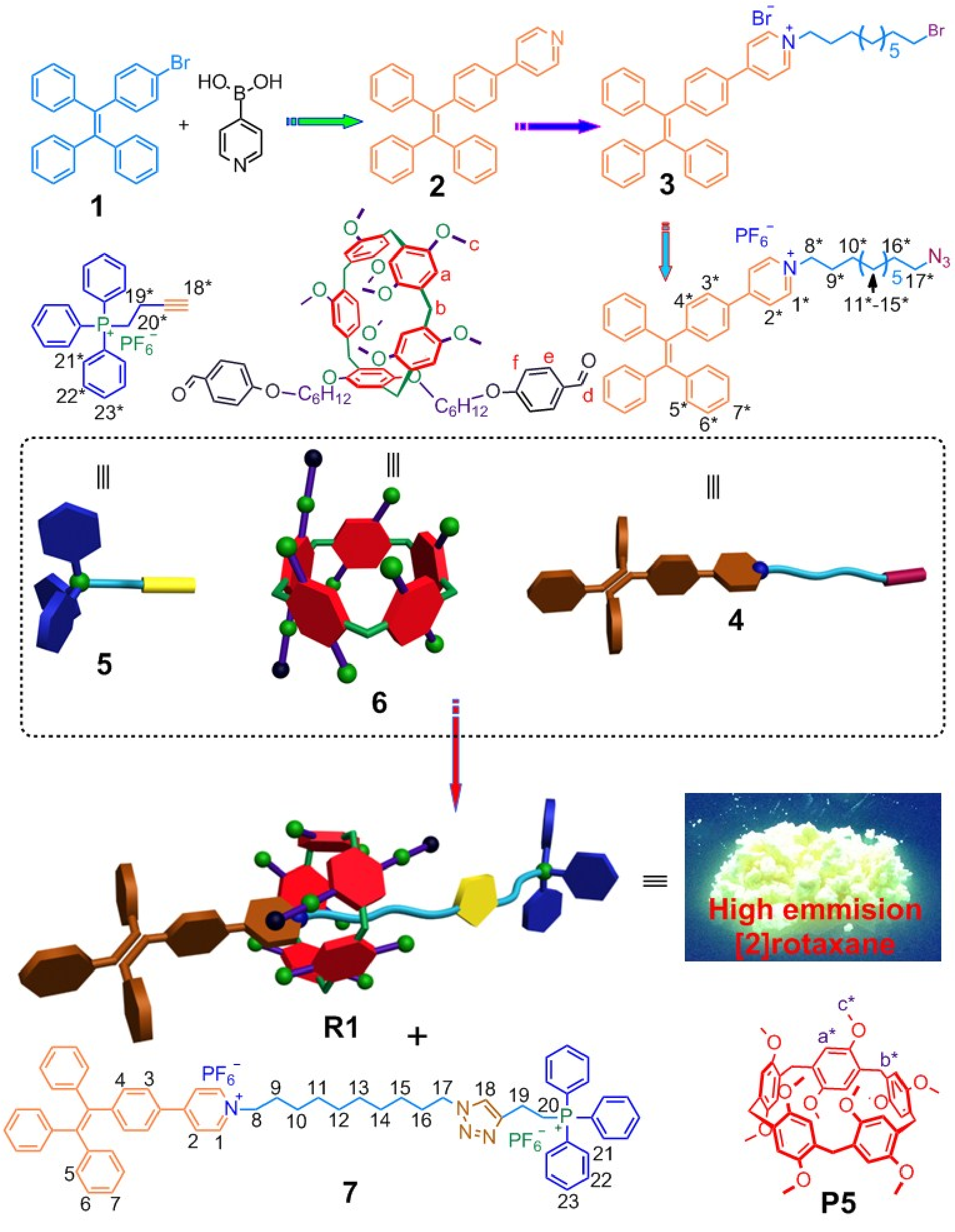

Scheme S1 Synthetic route to **R1**.

Synthesis of 2: 4-Pyridineboronic acid (1.23 g, 10.0 mmol) and K_2CO_3 (3.31 g, 24.0

mmol) were added to a solution of **1** (1.23 g, 3.00 mmol) in the mixture of toluene, enthol and water (100 mL, toluene/CH_3CH_2OH/H_2O = 7/2/1, *v*/*v*/*v*). The mixture was heated under nitrogen at reflux for 24 h. The organic solvent was removed and the water layer was extracted with dichloromethane (3 × 50 mL). The combined organic phase was washed with water (3 × 100 mL) and saturated NaCl solution (100 mL) and dried over anhydrous Na_2SO_4. After filtration and evaporation, the residue was purified by column chromatography on silica gel (dichloromethane/ethyl acetate, 20:1 *v*/*v*) to provide **2** as a light yellow solid (834 mg, 68%), m.p. 170.8−173.2 °C. The ^{1}H NMR spectrum of **2** is shown in Fig. S1. ^{1}H NMR (400 MHz, chloroform-*d*, room temperature) δ (ppm): 8.60 (s, 2H), 7.45 (d, J = 4 Hz, 2H), 7.39 (d, J = 4 Hz, 2H), 7.15−7.06 (m, 17H). The ^{13}C NMR spectrum of **2** is shown in Fig. S2. ^{13}C NMR (100 MHz, chloroform-*d*, room temperature) δ (ppm): 150.18, 144.88, 143.50, 143.45, 132.12, 131.38, 131.34, 131.31, 127.86, 127.82, 127.71, 127.67, 126.72, 126.66, 126.63, 126.17, 121.32. LRESIMS: *m*/*z* 410.4 [M + H]$^+$ (100%). HRESIMS: *m*/*z* calcd for [M + H]$^+$ $C_{31}H_{24}N$, 410.1909, found 410.1916, error 1.7 ppm.

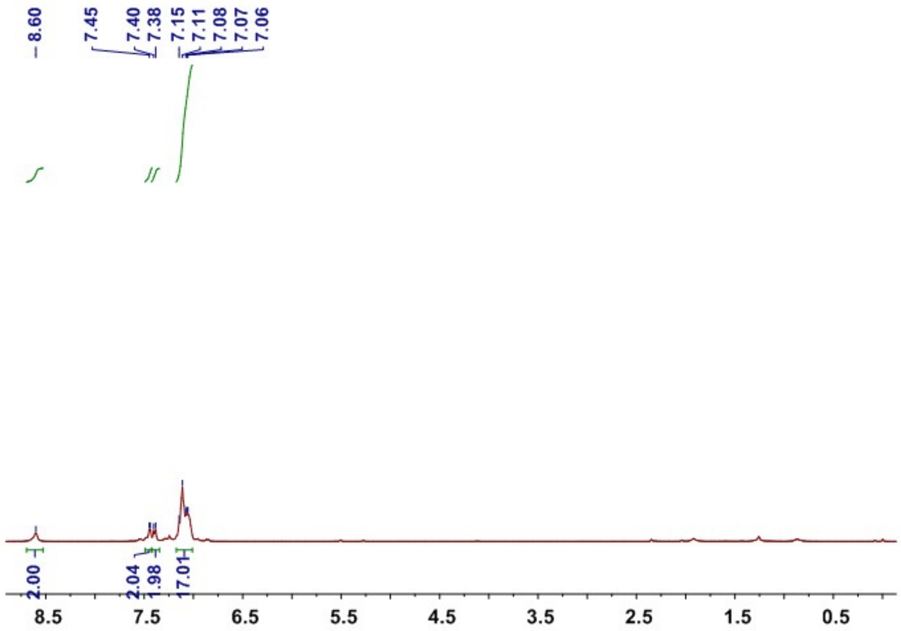

Fig. S1 ^{1}H NMR spectrum (400 MHz, chloroform-*d*, room temperature) of **2**.

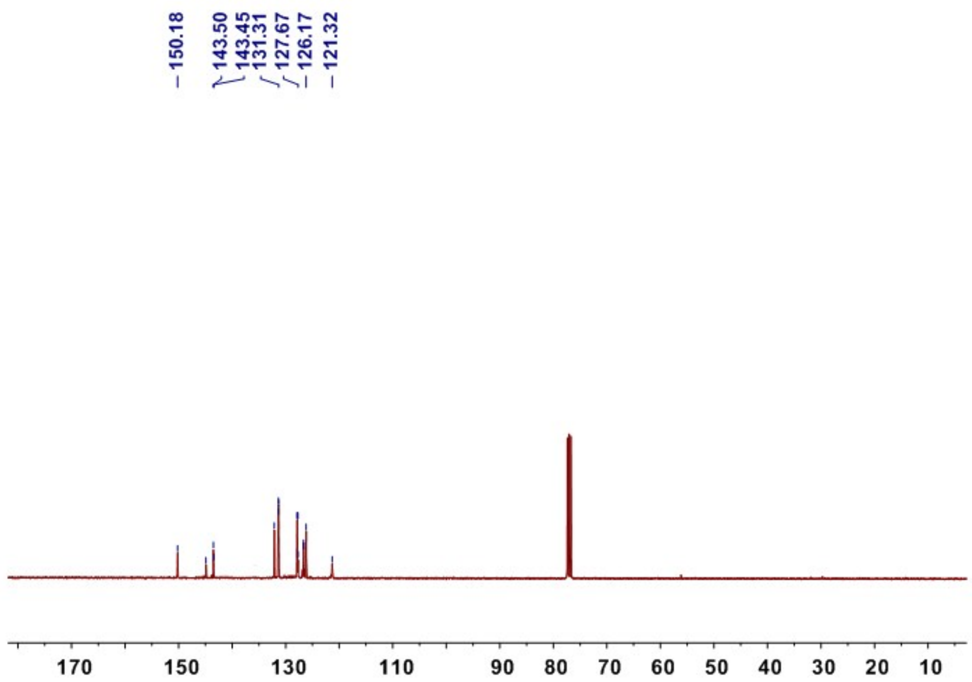

Fig. S2 ^{13}C NMR spectrum (100 MHz, chloroform-*d*, room temperature) of **2**.

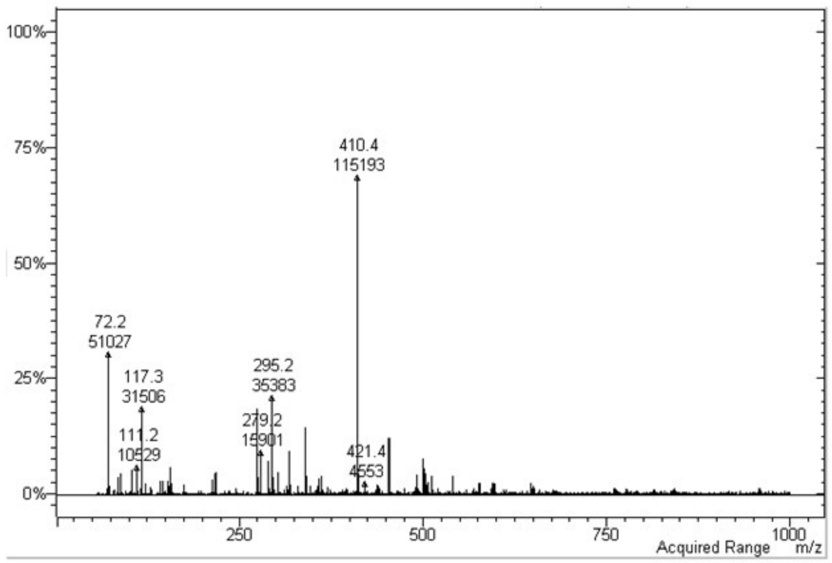

Fig. S3 Electrospray ionization mass spectrum of **2**. Assignment of the main peak: *m*/*z* 410.4 $[M + H]^+$ (100%).

Synthesis of 3: 1,10-Dibromodecane (12.0 g, 40.0 mmol) was added to a solution of **2** (1.63 g, 4.00 mmol) in CH_3CN (300 mL). The mixture was heated under nitrogen at reflux for 12 h. The cooled reaction mixture was evaporated under vacuum, and the

residue was purified by flash column chromatography (dichloromethane/methanol = 20/1 *v*/*v*) to yield **3** as a light yellow solid (2.23 g, 81%), m.p. 133.5−136.0 °C. The proton NMR spectrum of **3** is shown in Fig. S4. ^{1}H NMR (400 MHz, chloroform-*d*, room temperature) δ (ppm): 9.38 (d, J = 4 Hz, 2H), 8.24 (d, J = 4 Hz, 2H), 7.55 (d, J = 8 Hz, 2H), 7.23 (d, J = 4 Hz, 2H), 7.14−7.11 (m, 9H), 7.05−7.02 (m, 6H), 4.90 (t, J = 4 Hz, 2H), 3.39 (t, J = 8 Hz, 2H), 1.86−1.80 (m, 2H), 1.35−1.22 (m, 14H). The ^{13}C NMR spectrum of **3** is shown in Fig. S5. ^{13}C NMR (100 MHz, chloroform-*d*, room temperature) δ (ppm): 155.75, 148.91, 144.81, 143.38, 142.94, 142.77, 139.21, 131.28, 131.21, 130.97, 128.05, 128.02, 127.76, 127.21, 127.17, 126.98, 124.45, 61.10, 60.42, 58.36, 34.13, 32.75, 31.83, 28.06, 26.07, 18.44, 14.20. LRESIMS: *m*/*z* 628.4 [M − Br]$^{+}$ (22%). HRESIMS: *m*/*z* calcd for [M − Br]$^{+}$ $C_{41}H_{43}BrN$, 628.2579, found 628.2568, error −1.8 ppm.

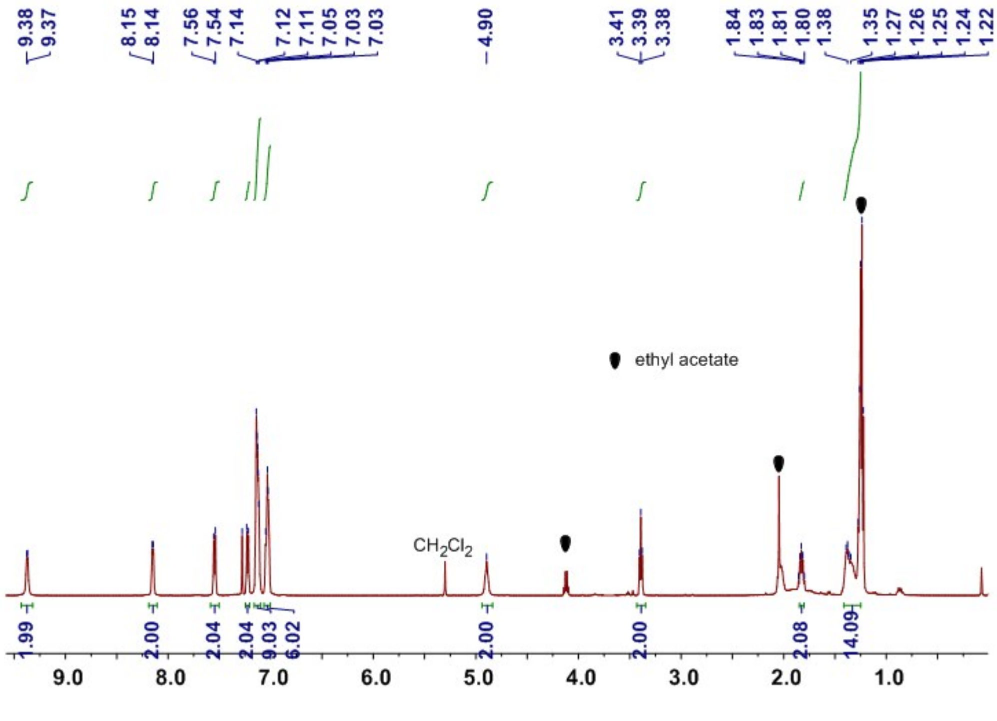

Fig. S4 ^{1}H NMR spectrum (400 MHz, chloroform-*d*, room temperature) of **3**.

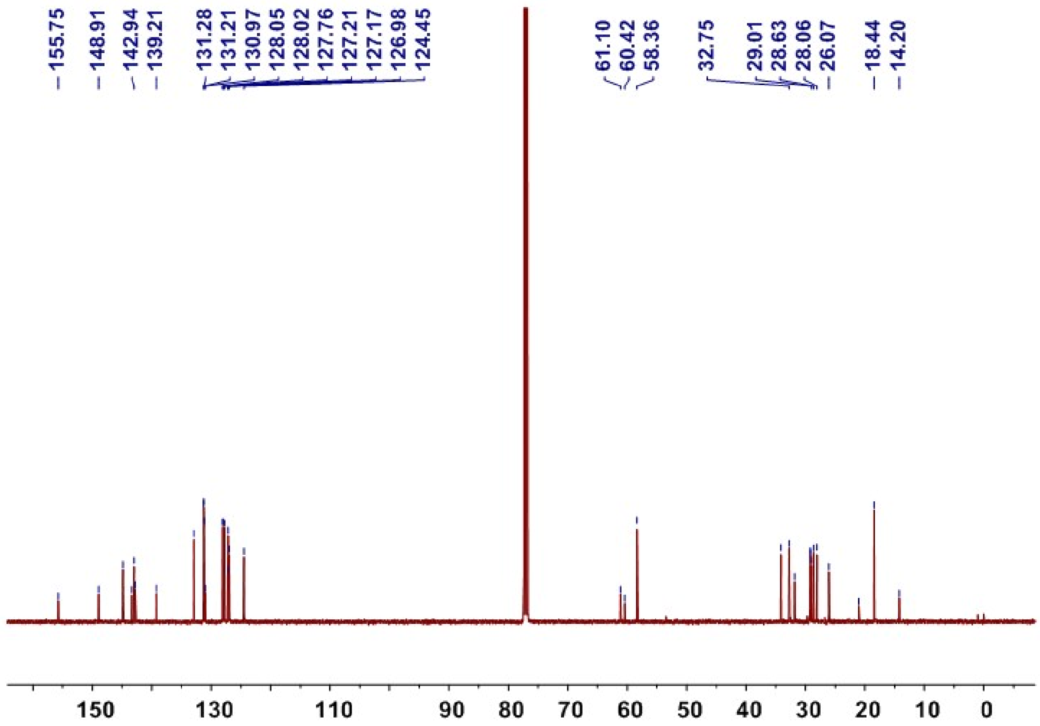

Fig. S5 ^{13}C NMR spectrum (100 MHz, chloroform-*d*, room temperature) of **3**.

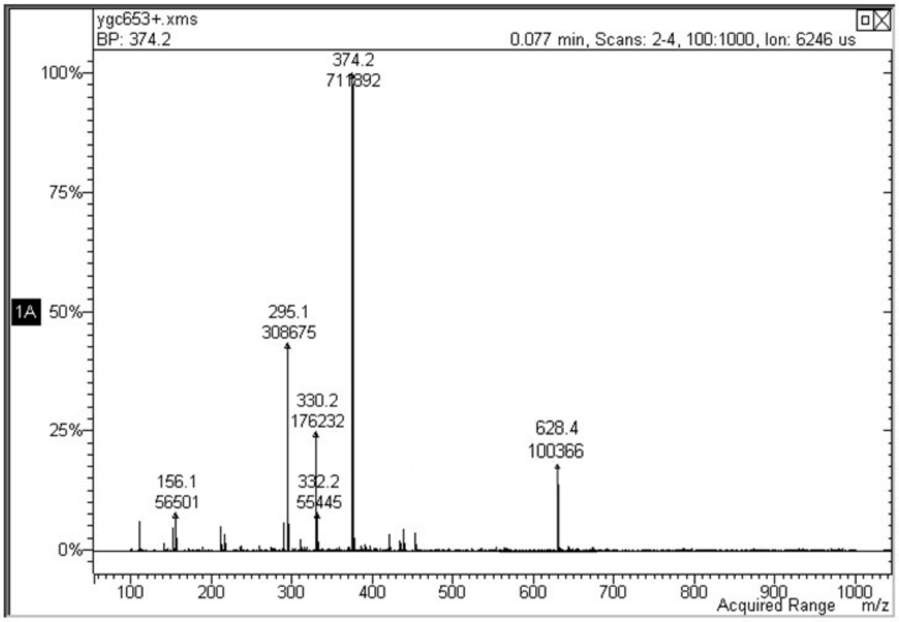

Fig. S6 Electrospray ionization mass spectrum of **3**. Assignment of the main peak: *m*/*z* 628.4 [M – Br]$^+$ (22%).

Synthesis of 4: A mixture of **3** (1.42 g, 2.00 mmol) and NaN_3 (1.63 g, 25.0 mmol) was

heated in the mixture of acetone (40 mL) and water (4 mL) at 50 °C for 12 h. KPF_6 (3.66 g, 20.0 mmol) was added into the mixture under stirring. The solution was concentrated under reduced pressure. The precipitate was filtered under suction and washed with water (5 × 20 mL) to give **4** as a light yellow solid (1.31 g, 91%), m.p. 126.4−128.6 °C. The proton NMR spectrum of **4** is shown in Fig. S7. ^{1}H NMR (400 MHz, chloroform-*d*, room temperature) δ (ppm): 9.02 (d, J = 8 Hz, 2H), 8.10 (d, J = 8 Hz, 2H), 7.53 (d, J = 8 Hz, 2H), 7.22 (d, J = 8 Hz, 2H), 7.15−7.12 (m, 9H), 7.06−7.01 (m, 6H), 4.75 (t, J = 8 Hz, 2H), 3.25 (t, J = 8 Hz, 2H), 2.05−1.96 (m, 2H), 1.61−1.54 (m, 2H), 1.34−1.26 (m, 12H). The ^{13}C NMR spectrum of **4** is shown in Fig. S8. ^{13}C NMR (100 MHz, chloroform-*d*, room temperature) δ (ppm): 155.95, 149.01, 144.33, 143.41, 142.95, 142.78, 139.20, 132.93, 131.30, 131.22, 130.87, 128.06, 128.03, 127.77, 127.23, 127.00, 124.44, 61.39, 51.45, 31.68, 29.26, 29.18, 29.01, 28.95, 28.80, 26.63, 26.15. LRESIMS: *m*/*z* 591.5 [M – PF_6]$^+$ (100%). HRESIMS: *m*/*z* calcd for [M − PF_6]$^+$ $C_{41}H_{43}N_4$, 591.3488, found 591.3471, error –2.9 ppm.

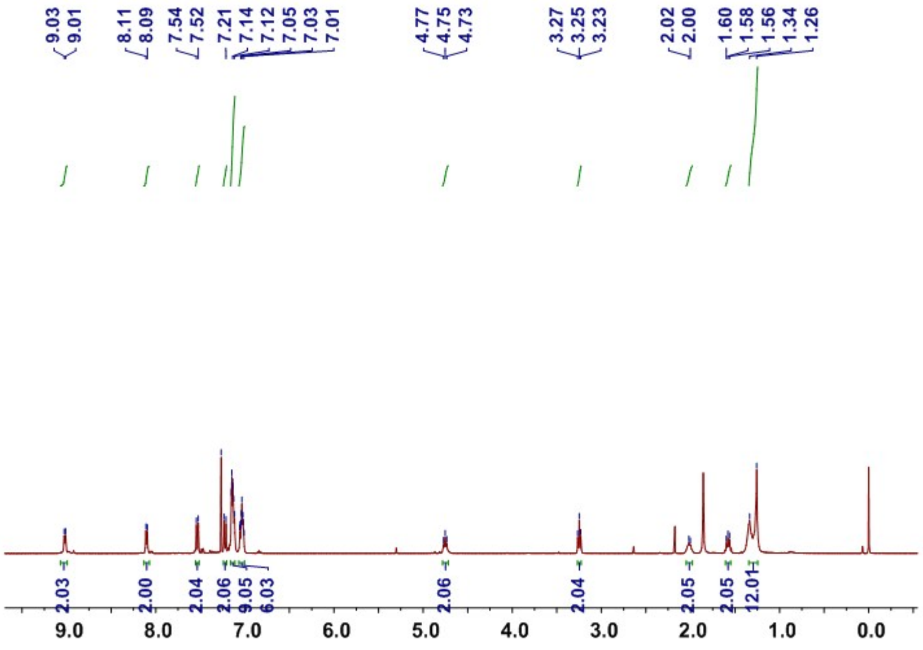

Fig. S7 ^{1}H NMR spectrum (400 MHz, chloroform-*d*, room temperature) of **4**.

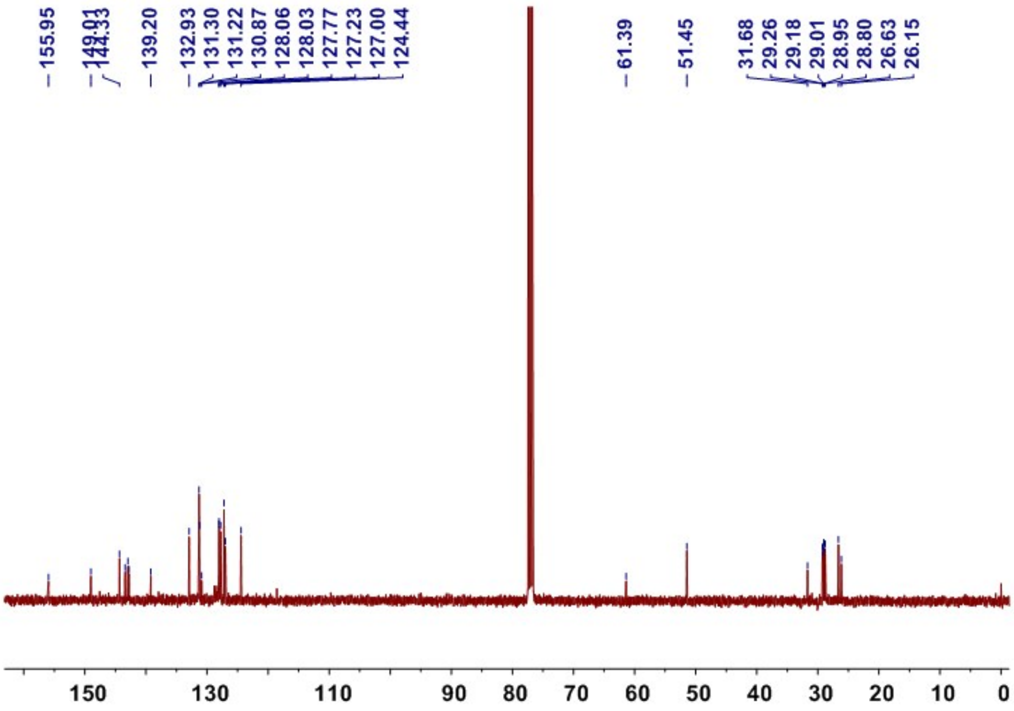

Fig. S8 ^{13}C NMR spectrum (100 MHz, chloroform-*d*, room temperature) of **4**.

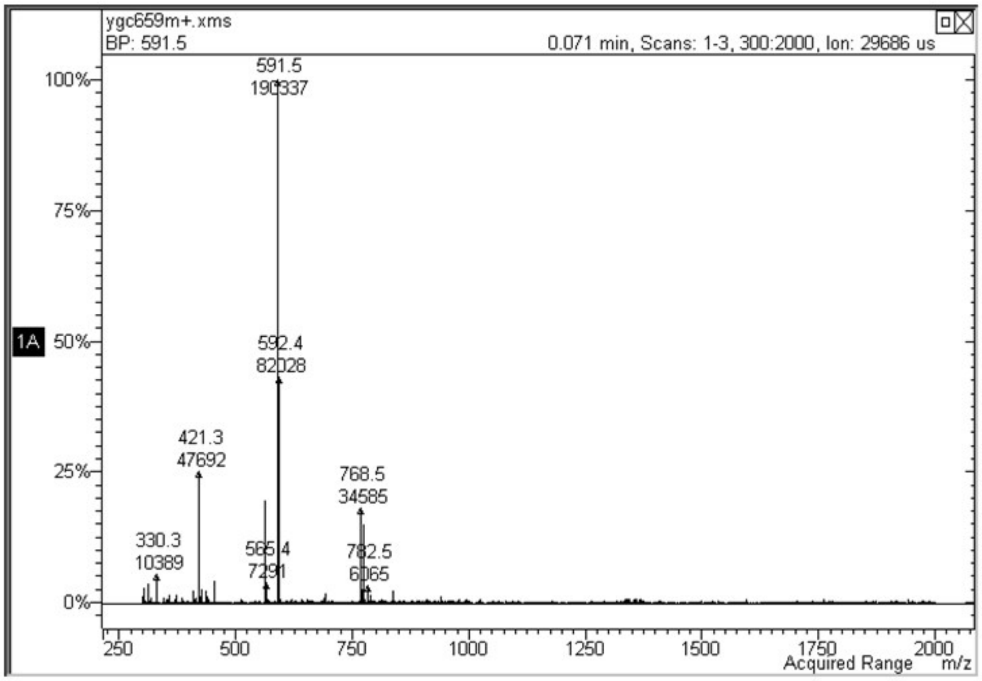

Fig. S9 Electrospray ionization mass spectrum of **4**. Assignment of the main peak: *m*/*z* 591.5 [M – PF_6]$^+$ (100%).

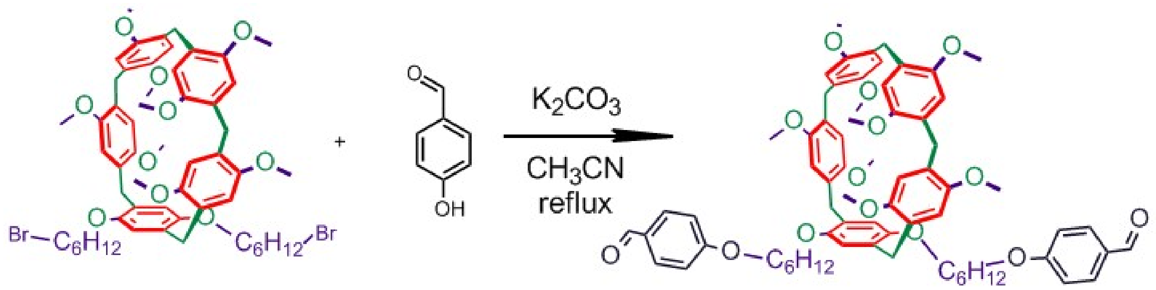

6a 6

Scheme S2 Synthetic route to **6**.

Synthesis of 6: 6a (2.09 g, 2.00 mmol) and K_2CO_3 (13.2 g, 98.0 mmol) were added to a solution of 4-hydroxybenzaldehyde (1.22 g, 10.0 mmol) in CH_3CN (300 mL). The mixture was heated in a three-necked flask under nitrogen atmosphere at reflux for 2 d. The cooled reaction mixture was filtered and washed with chloroform. The filtrate was evaporated under vacuum, and the residue was purified by flash column chromatography (dichloromethane/petroleum ether = 1:2, *v*/*v*) to yield **6** as a withe solid (1.99 g, 88%), m.p. 153.1−155.3 °C. The proton NMR spectrum of **6** is shown in Fig. S10. ^{1}H NMR (400 MHz, chloroform-*d*, room temperature) δ (ppm): 9.88 (s, 2H), 7.82 (d, J = 8 Hz, 4H), 6.93 (d, J = 8 Hz, 4H), 7.80–7.77 (m, 10H), 3.87–3.76 (m, 18H), 3.66–3.60 (m, 24H), 1.78–1.72 (m, 4H), 1.48–1.42 (m, 4H), 1.35–1.25 (m, 8H). The ^{13}C NMR spectrum of **6** is shown in Fig. S11. ^{13}C NMR (100 MHz, chloroform-*d*, room temperature) δ (ppm): 190.83, 150.70, 150.58, 150.49, 149.90, 131.95, 129.69, 128.44, 128.30, 128.26, 128.13, 116.37, 114.74, 113.93, 68.27, 55.90, 55.66, 53.29, 29.75, 29.50, 29.32, 29.23, 25.87, 25.83, 25.59. LRESIMS: *m*/*z* 1153.7 $[M + Na]^+$ (100%). HRESIMS: *m*/*z* calcd for $[M + Na]^+$ $C_{69}H_{78}O_{14}Na$, 1153.5289, found 1153.5311, error 1.9 ppm.

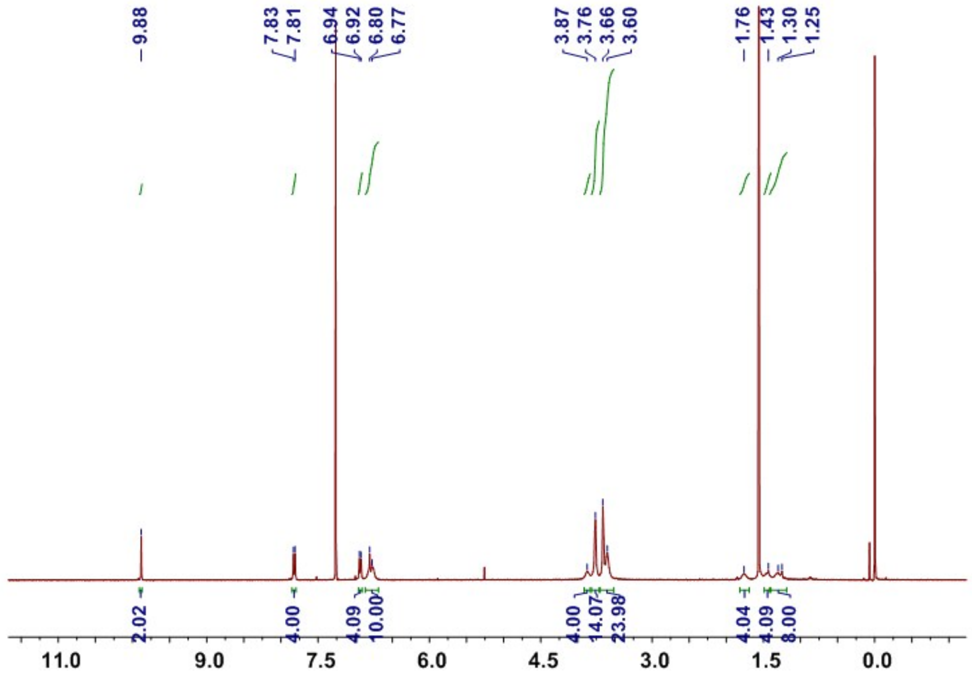

Fig. S10 ^{1}H NMR spectrum (400 MHz, chloroform-*d*, room temperature) of **6**.

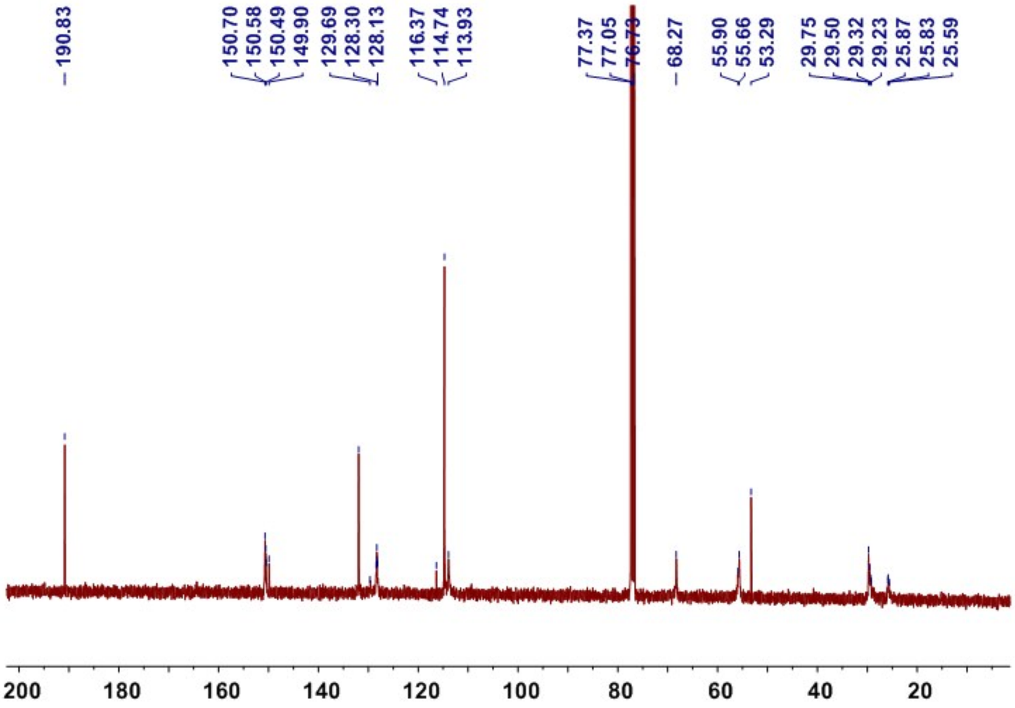

Fig. S11 ^{13}C NMR spectrum (100 MHz, chloroform-*d*, room temperature) of **6**.

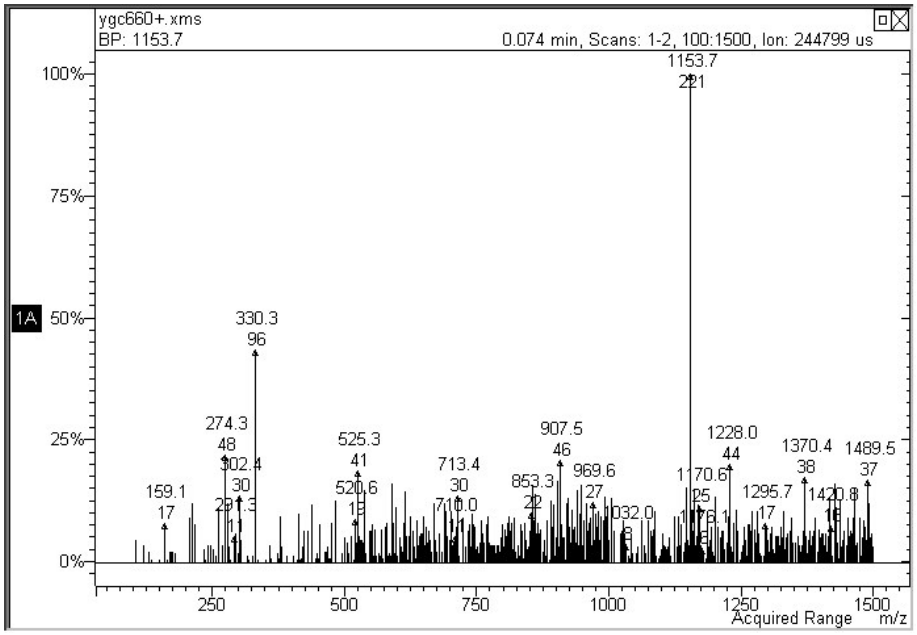

Fig. S12 Electrospray ionization mass spectrum of **6**. Assignment of the main peak: *m*/*z* 1153.7 [M + Na]$^+$ (100%).

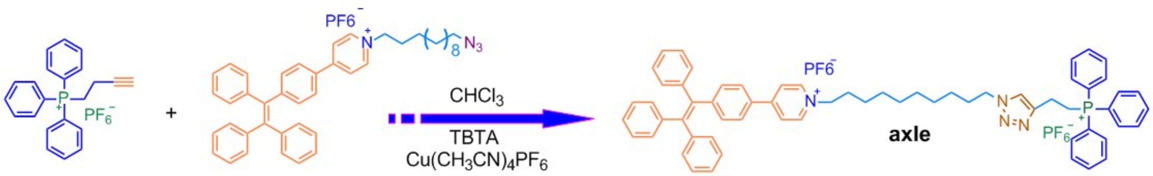

Scheme S3 Synthetic route to **7**.

Synthesis of 7: To a solution of **4** (736 mg, 1.00 mmol) and **5** (460 mg, 1.00 mmol) in chloroform (5 mL), tris[(1-benzyl-1H-1,2,3-triazol-4-yl)methyl]amine (TBTA, 106 mg, 0.200 mmol) was added. The reaction mixture was stirred at room temperature for 1 h. To the mixture, $Cu(CH_3CN)_4PF_6$ (74.4 mg, 0.200 mmol) was added, and the mixture was further stirred for 24 h. The resulting solution was concentrated under vacuo, and the residue was purified by flash column chromatography (dichloromethane/methanol = 10:1, *v*/*v*) to yield **7** as a light yellow solid (1.08 g, 90%), m.p. 88.5−91.2 °C. The proton NMR spectrum of **7** is shown in Fig. S13. ^{1}H NMR (400 MHz, chloroform-*d*, room temperature) δ (ppm): 8.86 (d, J = 4 Hz, 2H), 8.08 (d, J = 4 Hz, 2H), 8.02 (s, 1H), 7.82–7.79 (m, 3H), 7.74–7.67 (m, 12H), 7.53 (d, J = 4 Hz, 2H), 7.34 (d, J = 4 Hz, 2H), 7.21 (d, J = 4 Hz, 2H), 7.14–7.11 (m, 8H), 7.05–7.01 (m, 5H), 4.57 (t, J = 8 Hz, 2H), 4.29 (t, J =

8 Hz, 2H), 3.65–3.59 (m, 2H), 3.21–3.15 (m, 2H), 1.99 (t, J = 4 Hz, 2H), 1.88 (t, J = 4 Hz, 2H), 1.35–1.24 (m, 12H). The ^{13}C NMR spectrum of **7** is shown in Fig. S14. ^{13}C NMR (100 MHz, chloroform-d, room temperature) δ (ppm): 155.42, 148.58, 145.10, 143.02, 142.83, 139.30, 135.27, 133.81, 133.71, 132.80, 131.31, 131.22, 130.65, 129.09, 128.70, 128.47, 128.02, 127.74, 127.16, 126.92, 124.46, 118.14, 117.28, 60.75, 54.15, 50.26, 31.64, 29.71, 28.46, 28.34, 28.19, 25.87, 25.50, 22.77, 19.43. LRESIMS: m/z 453.7 [M − 2PF$_6$]$^{2+}$ (100%). HRESIMS: m/z calcd for [M − 2PF$_6$]$^{2+}$ $C_{63}H_{63}N_4P$, 453.5869, found 453.5877, error 1.7 ppm.

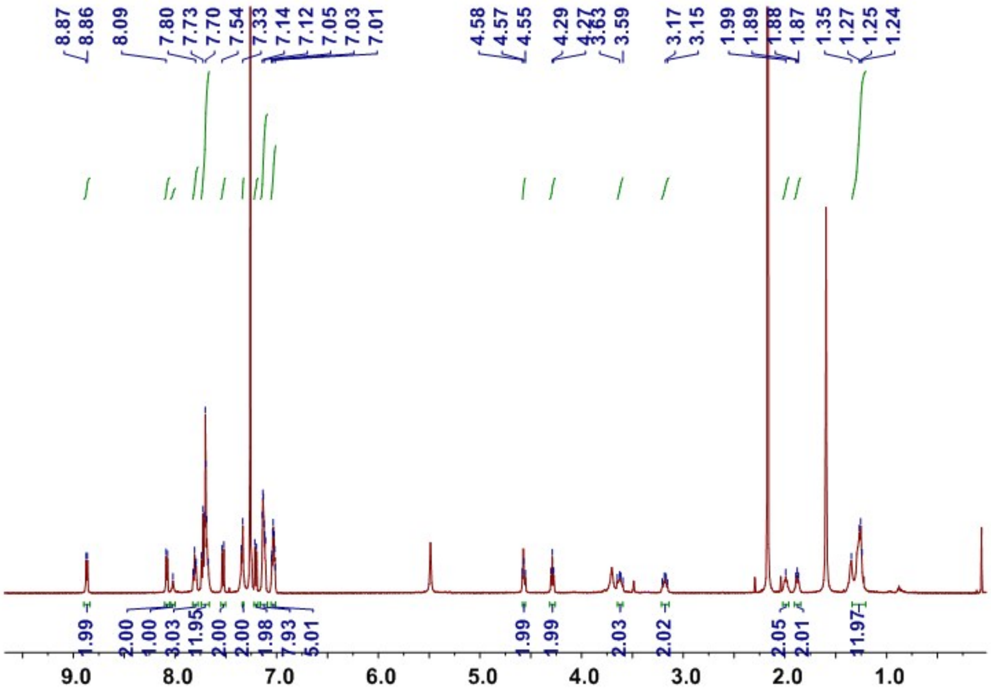

Fig. S13 ^{1}H NMR spectrum (400 MHz, chloroform-d, room temperature) of **7**.

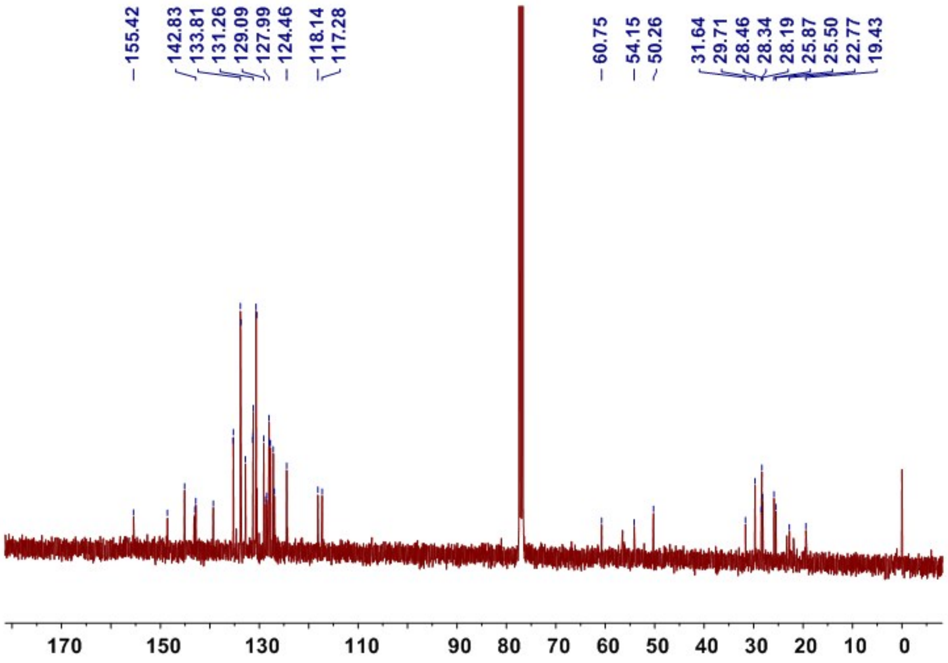

Fig. S14 ^{13}C NMR spectrum (100 MHz, chloroform-*d*, room temperature) of **7**.

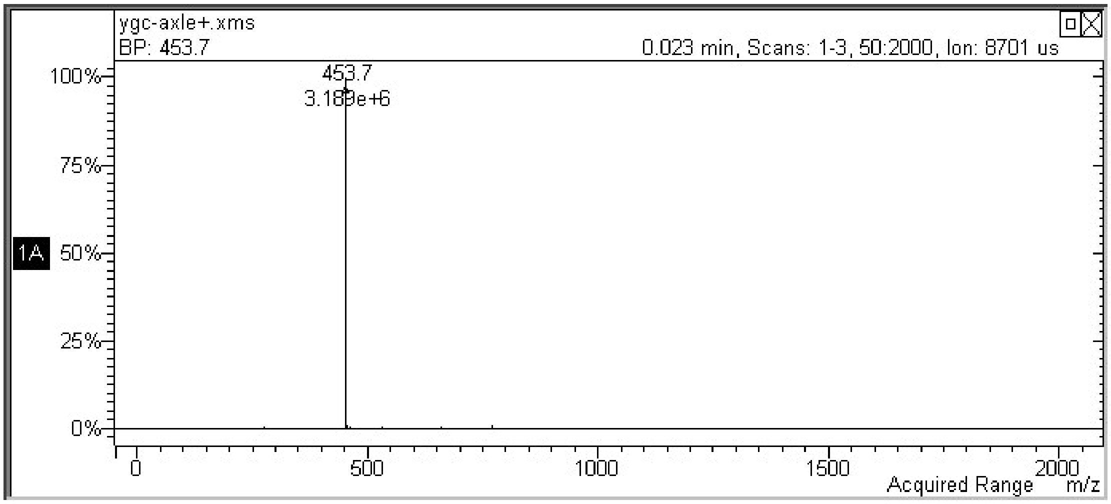

Fig. S15 Electrospray ionization mass spectrum of **7**. Assignment of the main peak: *m*/*z* 453.7 [M − $2PF_6]^{2+}$ (100%).

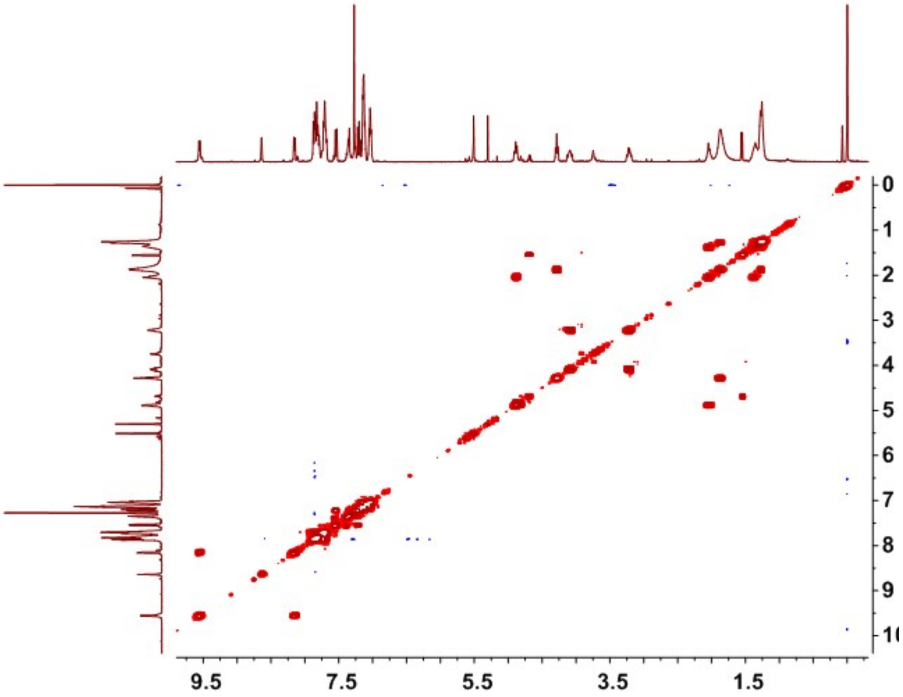

Fig. S16 2D COSY spectrum (chloroform-*d*, room temperature) of **7**.

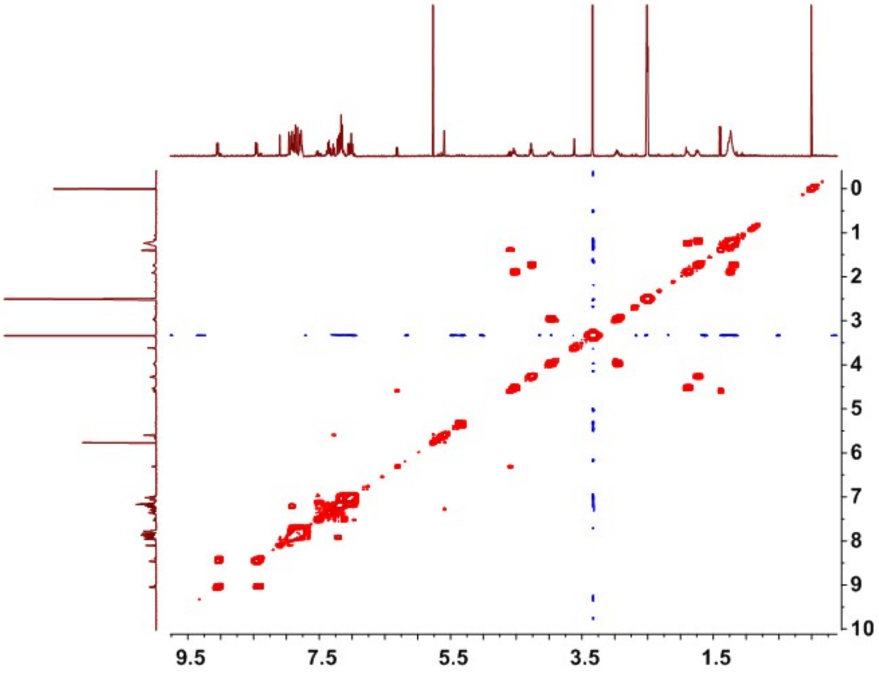

Fig. S17 2D COSY spectrum (DMSO-d_6, room temperature) of **7**.

Synthesis of R1: To a solution of **4** (368 mg, 0.500 mmol), **6** (1.13 g, 1.00 mmol) and stopper **5** (460 mg, 1.00 mmol) in chloroform (5 mL), tris[(1-benzyl-1H-1,2,3- triazol-4-yl)methyl]amine (TBTA, 106 mg, 0.200 mmol) was added. The reaction mixture was stirred at room temperature for 1 h. To the mixture, $Cu(CH_3CN)_4PF_6$ (74.4 mg, 0.200 mmol) was added, and the mixture was further stirred for 24 h. The resulting solution was concentrated under vacuo, and the residue was purified by flash column chromatography (dichloromethane/petroleum ether, 1:2 *v*/*v*) to yield **R1** as a light yellow solid (326 mg, 28%), m.p. 177.1−179.3 °C. The proton NMR spectrum of **R1** is shown in Fig. S18. ^{1}H NMR (400 MHz, chloroform-*d*, room temperature) δ (ppm): 9.84 (s, 2H), 8.00 (s, 1H), 7.85–7.82 (m, 6H), 7.80 (d, *J* = 4 Hz, 4H), 7.78 (d, *J* = 4 Hz, 4H), 7.74–7.70 (m, 15H), 7.38 (d, *J* = 4 Hz, 4H), 7.15–7.10 (m, 9H), 7.07–7.03 (m, 4H), 6.99–6.94 (m, 6H), 6.73 (s, 2H), 6.66 (s, 2H), 6.64 (s, 2H), 6.62 (s, 2H), 6.60 (s, 2H), 4.31 (t, *J* = 8 Hz, 4H), 4.09 (t, *J* = 8 Hz, 4H), 4.02 (t, *J* = 8 Hz, 4H), 3.78–3.75 (m, 12H), 3.70–3.65 (m, 12H), 3.56–3.50 (m, 10H), 3.16–3.11 (m, 4H), 1.95–1.90 (m, 4H), 1.89–1.86 (m, 4H), 1.78–1.76 (m, 4H), 1.65–1.62 (m, 4H), 1.49–1.46 (m, 4H), 1.39–1.31 (m, 8H), 1.95–1.90 (m, 4H), –1.04 (m, 2H), –1.13 (m, 2H). The ^{13}C NMR spectrum of **R1** is shown in Fig. S19. ^{13}C NMR (100 MHz, chloroform-*d*, room temperature) δ (ppm): 191.18, 163.54, 149.69, 142.44, 135.02, 133.76, 133.66, 132.11, 131.75, 130.66, 130.54, 130.29, 130.16, 129.50, 129.47, 128.47, 128.15, 128.10, 128.04, 127.97, 127.92, 127.85, 127.77, 127.74, 127.57, 127.05, 126.91, 123.51, 121.62, 118.52, 117.66, 114.78, 114.75, 113.23, 67.82, 56.04, 56.00, 55.67, 55.58, 55.46, 55.33, 55.27, 55.09, 54.88, 49.21, 29.93, 29.81, 29.76, 29.46, 29.22, 29.19, 28.91, 28.84, 28.80, 28.40, 28.19, 28.17, 25.46, 25.31, 18.47. LRESIMS: *m*/*z* 1019.5 $[M - 2PF_6]^{2+}$ (100%). HRESIMS: *m*/*z* calcd for $[M - 2PF_6]^{2+}$ $C_{132}H_{141}N_4O_{14}P$, 1018.5091, found 1018.5117, error 2.6 ppm.

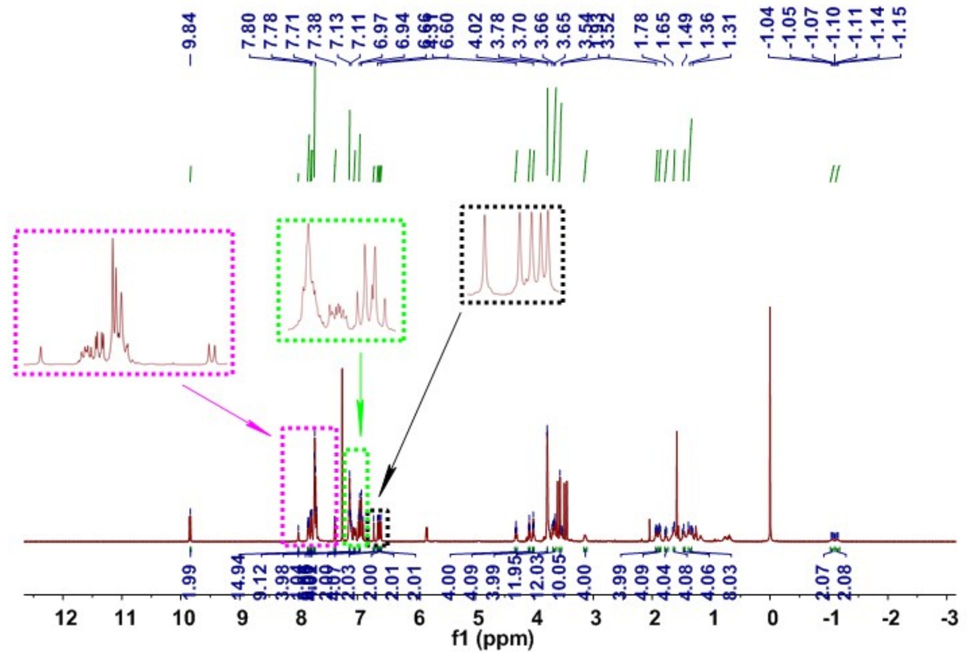

Fig. S18 ^{1}H NMR spectrum (400 MHz, chloroform-*d*, room temperature) of **R1**.

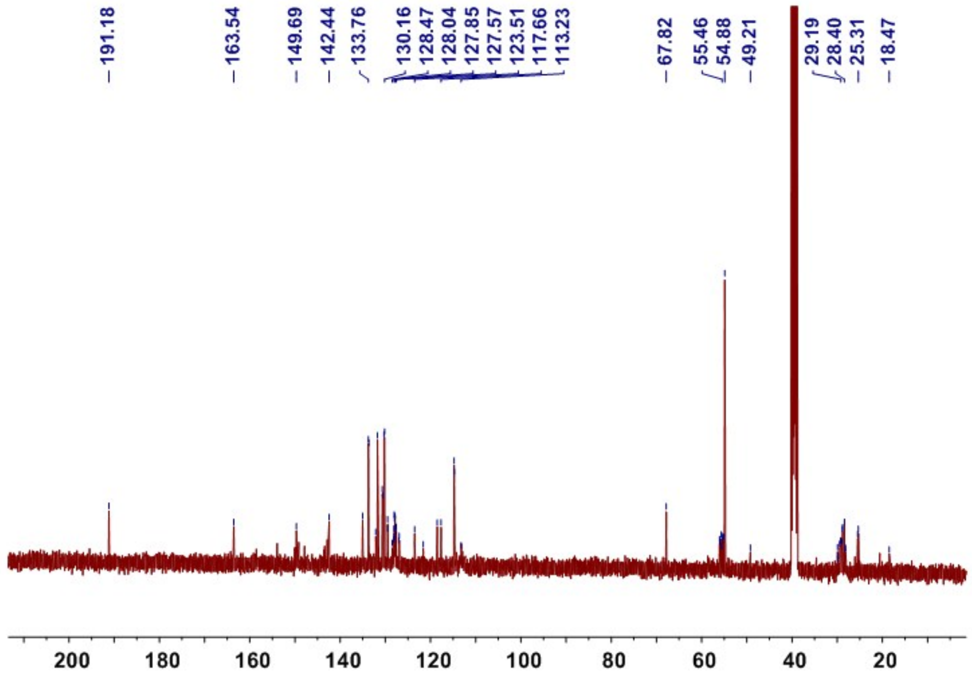

Fig. S19 ^{13}C NMR spectrum (100 MHz, chloroform-*d*, room temperature) of **R1**.

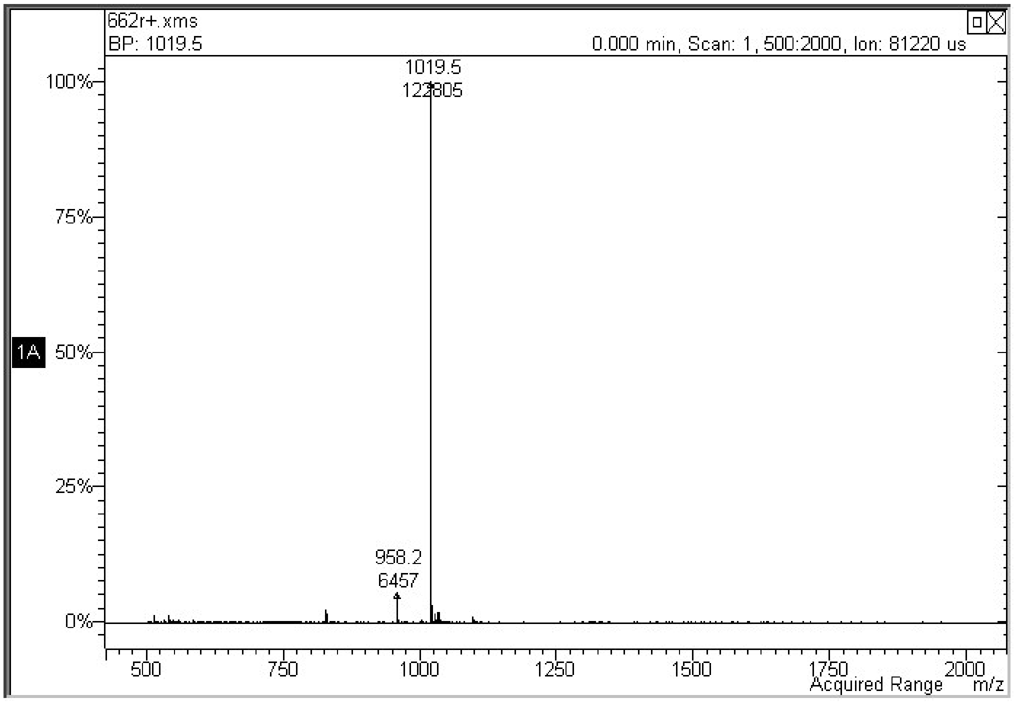

Fig. S20 Electrospray ionization mass spectrum of **R1**. Assignment of the main peak: *m*/*z* 1019.5 $[M - 2PF_6]^{2+}$ (100%).

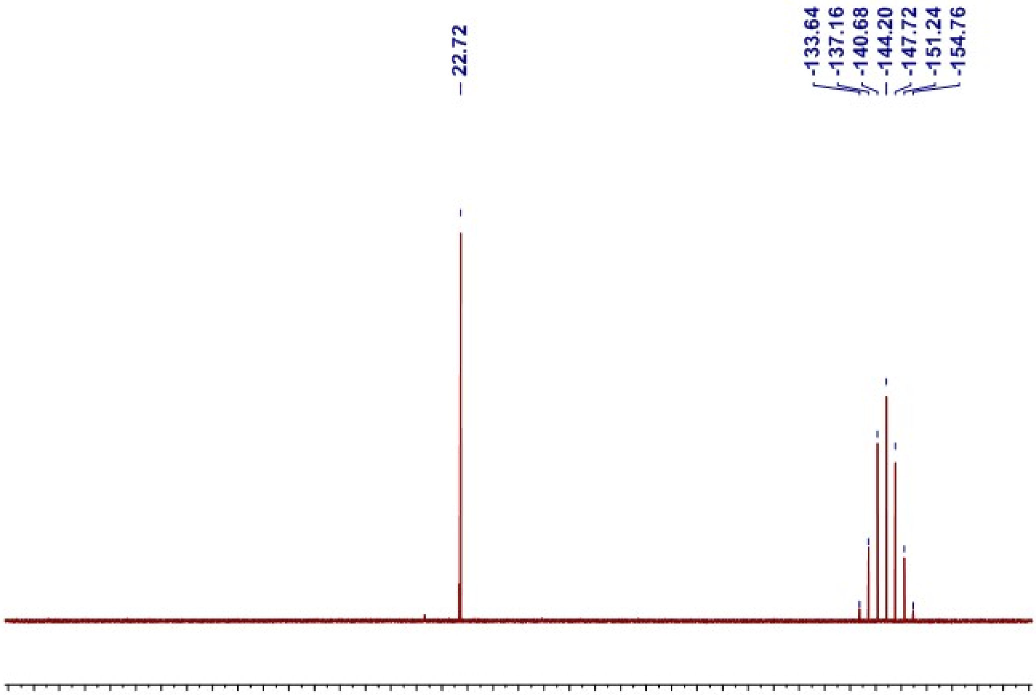

Fig. S21 ^{31}P NMR spectrum (202.3 MHz, chloroform-*d*, room temperature) of **R1**.

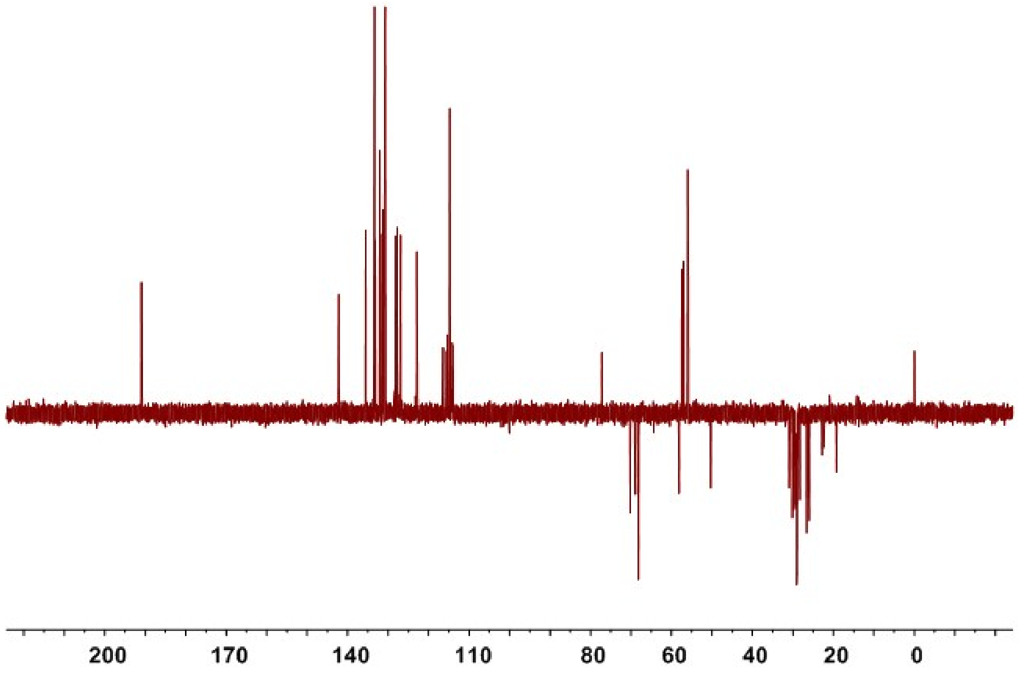

Fig. S22 DEPT135 spectrum (chloroform-*d*, room temperature) of **R1**.

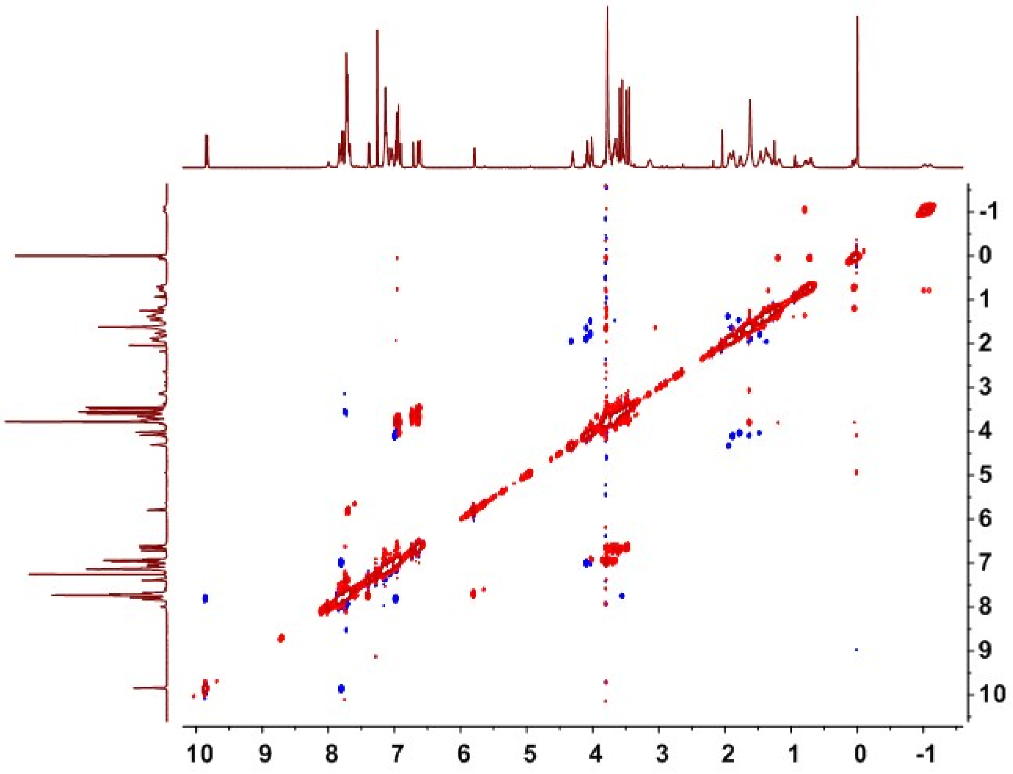

Fig. S23 2D COSY spectrum (chloroform-*d*, room temperature) of **R1**.

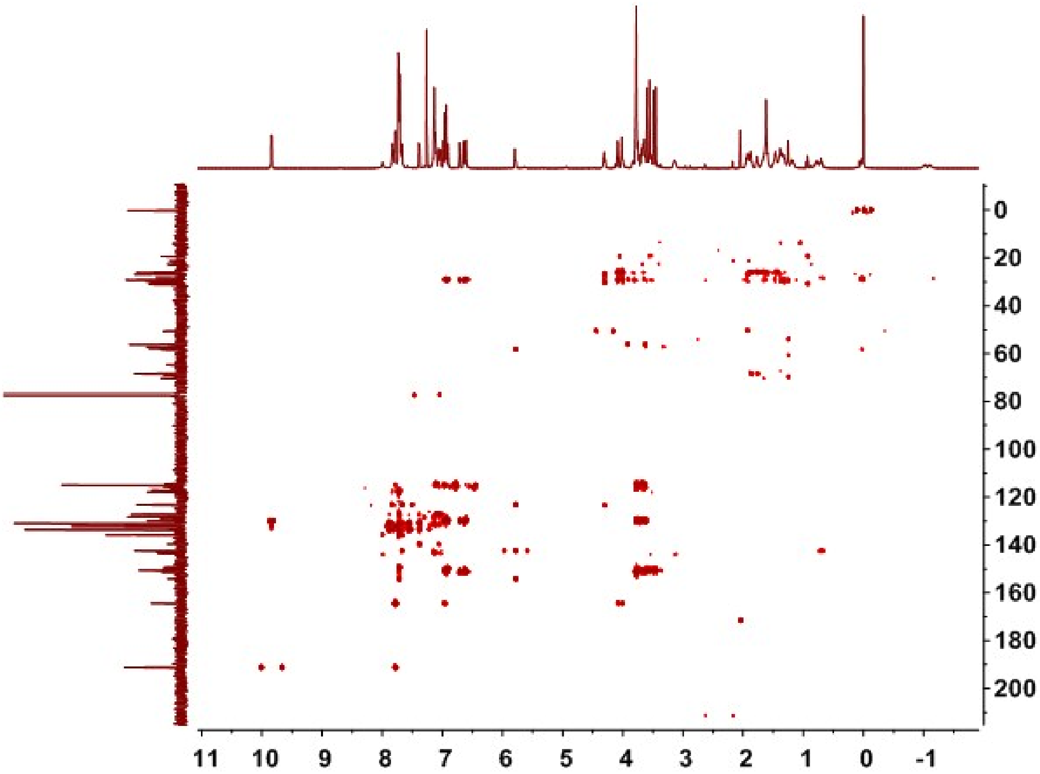

Fig. S24 HMBC spectrum (chloroform-*d*, room temperature) of **R1**.

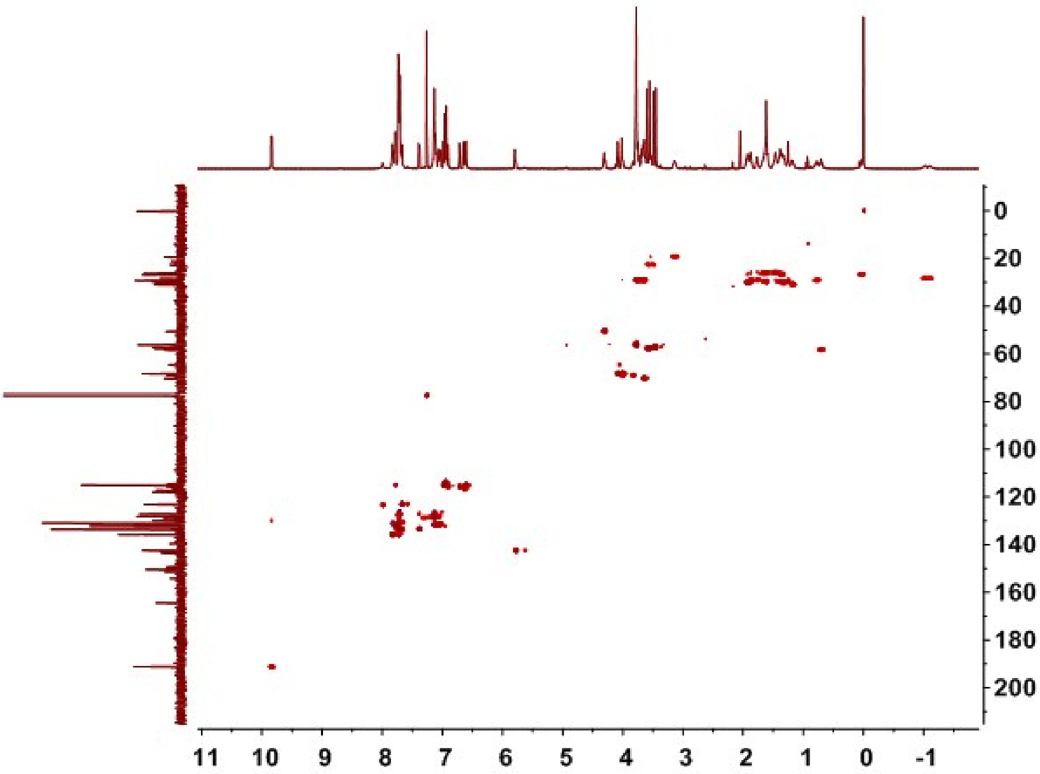

Fig. S25 HSQC spectrum (chloroform-*d*, room temperature) of **R1**.

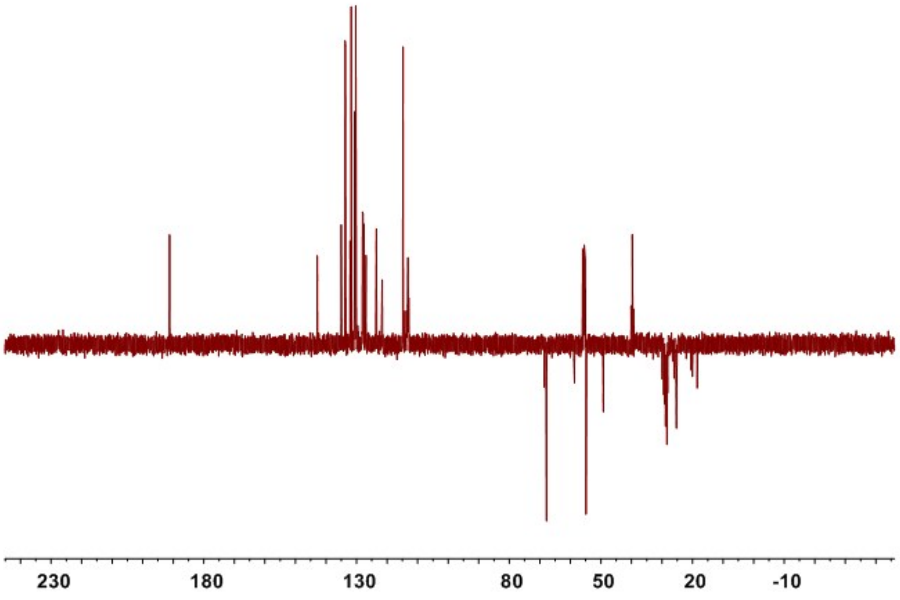

Fig. S26 DEPT135 spectrum (DMSO-d_6, room temperature) of **R1**.

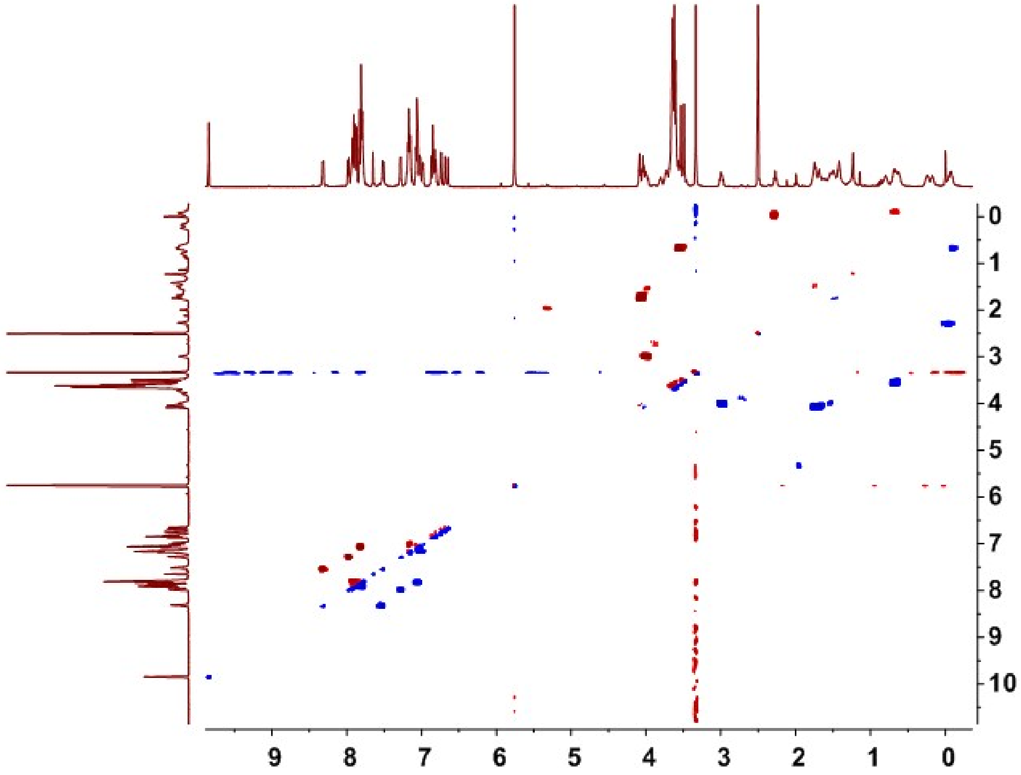

Fig. S27 2D COSY spectrum (DMSO-d_6, room temperature) of **R1**.

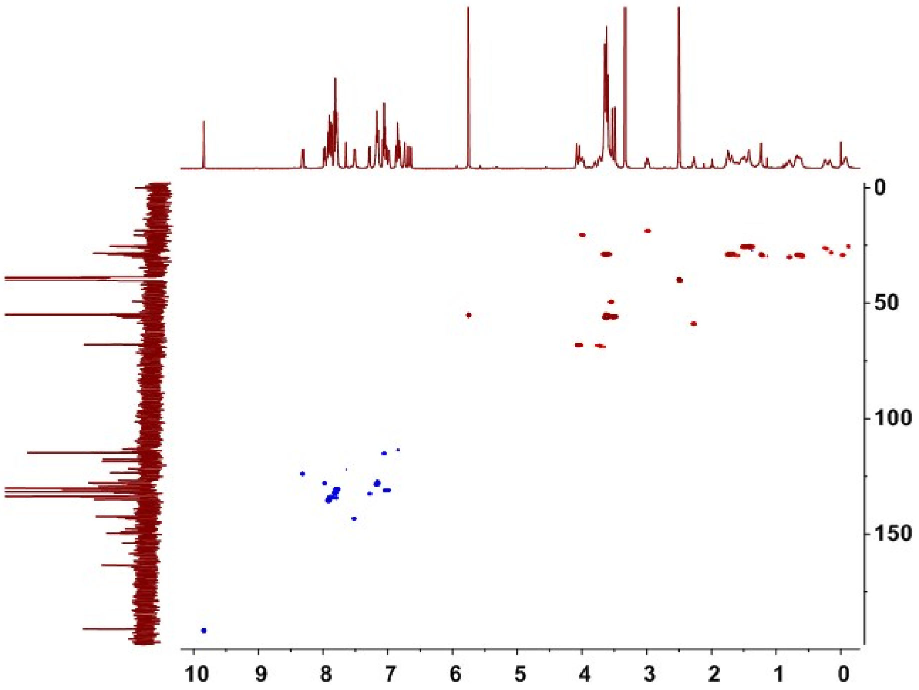

Fig. S28 HMBC spectrum (DMSO-d_6, room temperature) of **R1**.

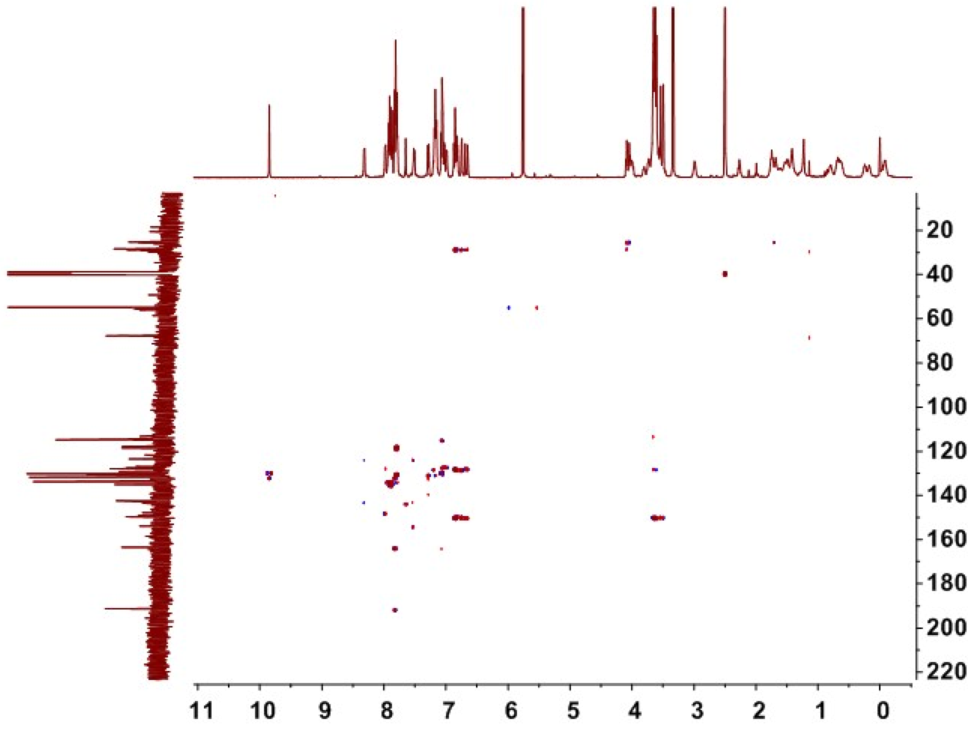

Fig. S29 HSQC spectrum (DMSO-d_6, room temperature) of **R1**.

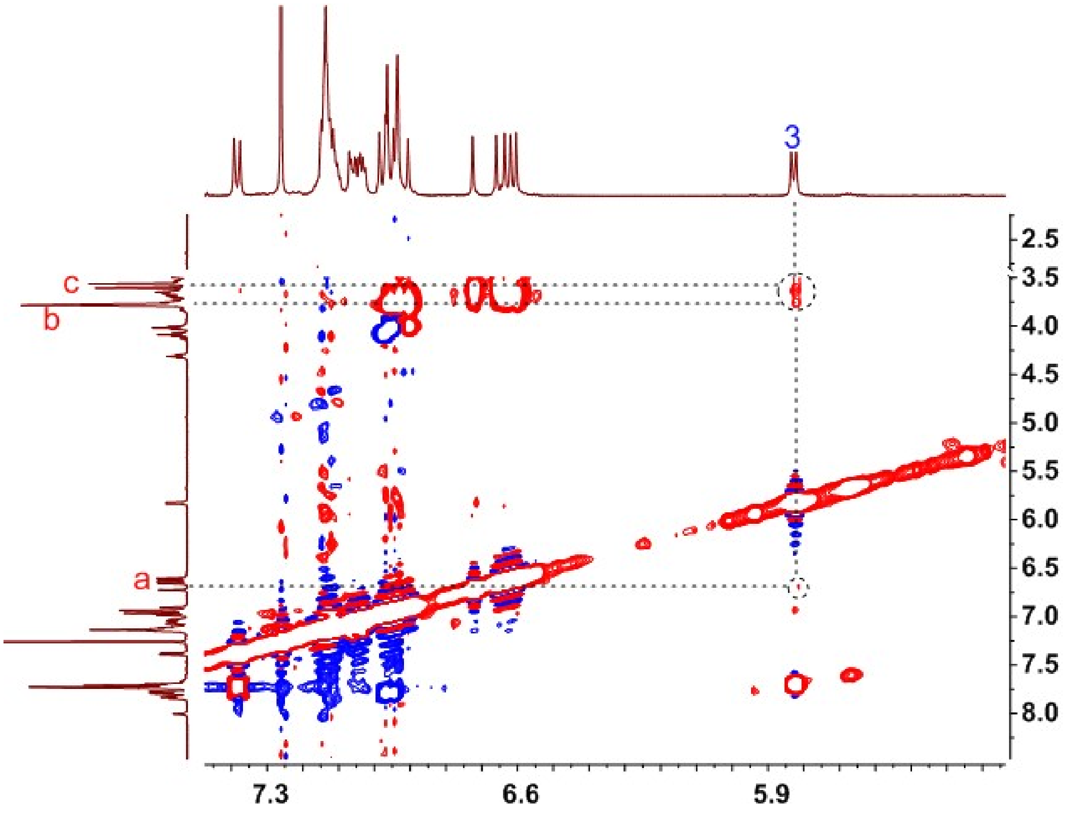

Fig. S30 Partial 2D NOESY spectrum (chloroform-*d*, room temperature) of **R1**.

*3. Host–guest complexations between **4**, **5**, and **P5***

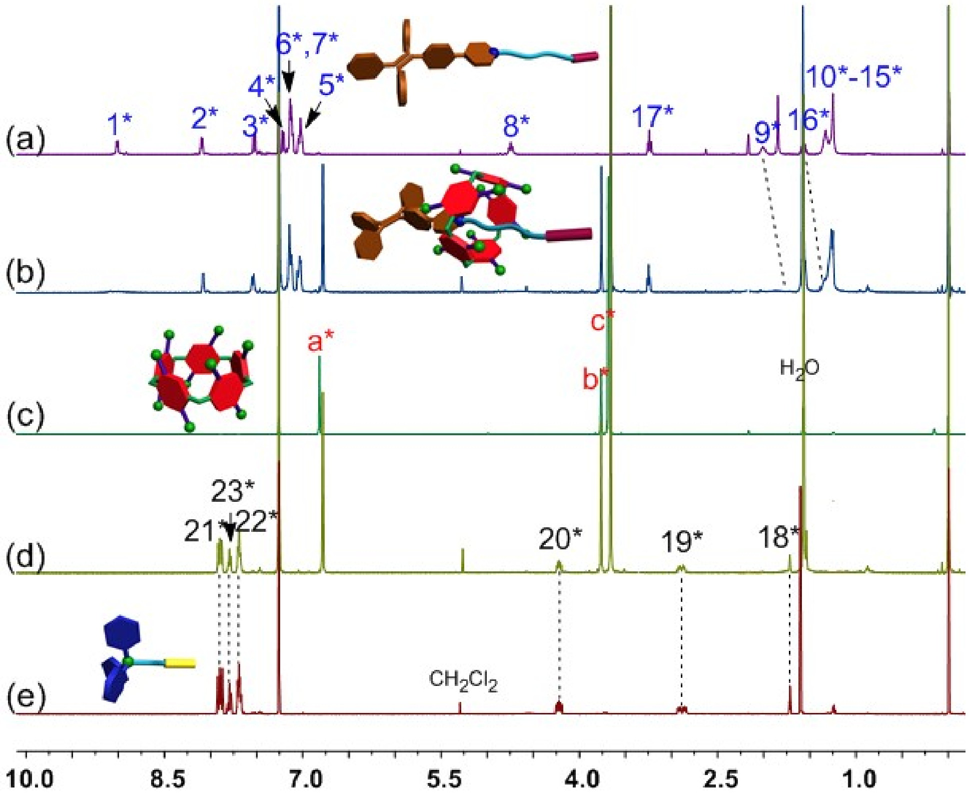

Fig. S31 ^{1}H NMR spectra (400 MHz, chloroform-*d*, 295 K): (a) **4** (2.00 mM); (b) **P5** (2.00 mM) and **4** (2.00 mM); (c) **P5** (2.00 mM); (d) **P5** (2.00 mM) and **5** (2.00 mM); (e) **5** (2.00 mM).

The host–guest interactions among **4**, **5**, and **6** were investigated through ^{1}H NMR spectroscopy (Fig. S31) by employing 1,4-dimethoxypillar[5]arene (**P5**) as a model host. The ^{1}H NMR spectrum of an equimolar solution of **P5** and **4** in chloroform-*d* contains only one set of peaks (Fig. S31b), demonstrating fast-exchange complexation between **P5** and **4** on the ^{1}H NMR time scale. Compared with the spectrum of free **4** (Fig. S31a), the resonance peaks related to protons H_{2*}, H_{9*} and $H^{1}{}_{0*}$ displayed upfield shifts ($\Delta\delta = -0.02$, -0.31 and -0.24 ppm for H_{2*}, H_{9*} and H_{10*}, respectively) in the presence of an equivalent amount of **P5**. Moreover, extensive broadening effects were observed for the peaks corresponding to protons H_{1*}, H_{8*}, H_{9*} and H_{16*} on **4** due to complexation dynamics. For example, the signals of protons H_{1*} and H_{8*} disappeared upon complexation between **P5** and **4**. The reason was that these protons were shielded by the electron-rich cyclic structure upon formation of a threaded structure between **P5** and **4**. On the other hand, the

signals related to the protons on **P5** also exhibited slight chemical shift changes due to the interactions between **P5** and **4**. The resonance peaks related to aromatic protons H_{a*} and methylene protons H_{c*} shifted downfield ($\Delta\delta$ = 0.03 and 0.03 ppm for H_{a*} and H_{c*}, respectively). The driving forces for the complexation between **P5** and **4** were the cooperativity of cation•••π and multiple C-H•••π interactions. It should be noted that no chemical shift changes were observed for the peaks related to the protons on **5** upon addition of **P5** (Fig. S31d), indicating weak or no host–guest complexation.

To determine the stoichiometries and association constants for the complexations between **P5** and the guests (**4** and **5**), ^{1}H NMR titrations were done with solutions which had a constant concentration of the guest (**4** or **5**) (2.00 mM) and varying concentrations of host **P5**. By a non-linear curve-fitting method, the association constant (K_a) of **P5⊃4** (or **5**) was estimated. By a mole ratio plot, 1:1 stoichiometry was obtained.

The non-linear curve-fitting was based on the equation:[S3]

$$\Delta\delta = (\Delta\delta_\infty/[G]_0)\ (0.5[H]_0 + 0.5([G]_0+1/K_a) - (0.5\ ([H]_0{}^2 + (2[H]_0(1/K_a - [G]_0)) + (1/K_a + [G]_0)^2)\ ^{0.5}))\ \text{(Eq. S1)}$$

Where $\Delta\delta$ is the chemical shift change of H_3 on **4** (or H_{13} on **5**) at $[H]_0$, $\Delta\delta_\infty$ is the chemical shift change of H_3 (or H_{13}) when **4** (or **5**) is completely complexed, $[H]_0$ is the initial concentration of **P5**, and $[G]_0$ is the fixed initial concentration of the guest.

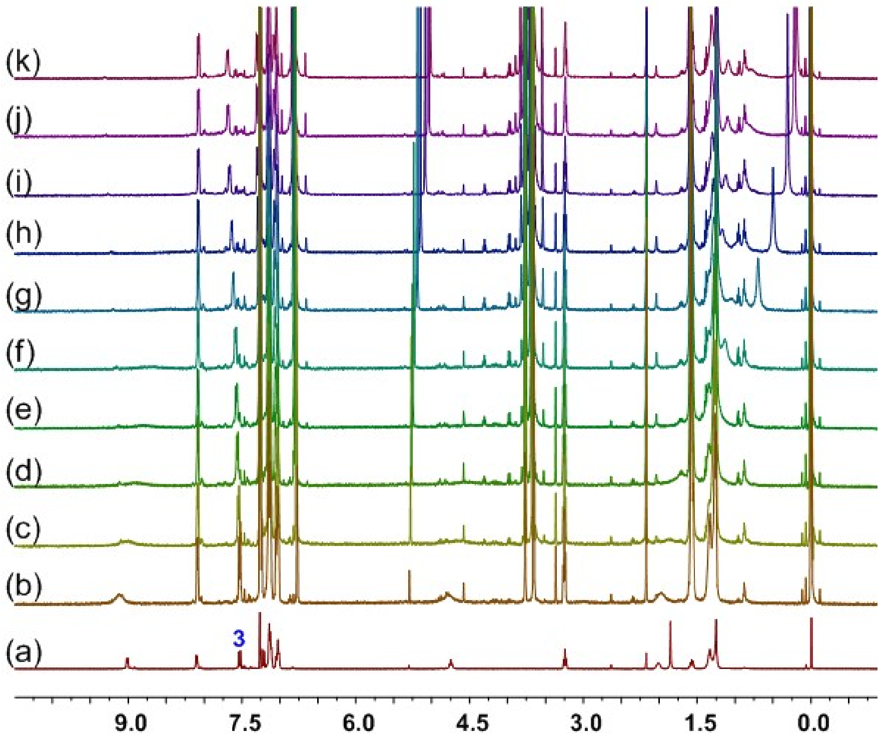

Fig. S32 Partial ^{1}H NMR spectra (500 MHz, chloroform-*d*, room temperature) of **4** at a concentration of 2.00 mM upon addition of **P5**: (a) 0 mM, (b) 0.200 mM, (c) 0.450 mM, (d) 1.31 mM, (e) 1.71 mM, (f) 2.14 mM, (g) 4.52 mM, (h) 5.24 mM, (i) 7.62 mM, (j) 11.1 mM, and (k) 13.6 mM.

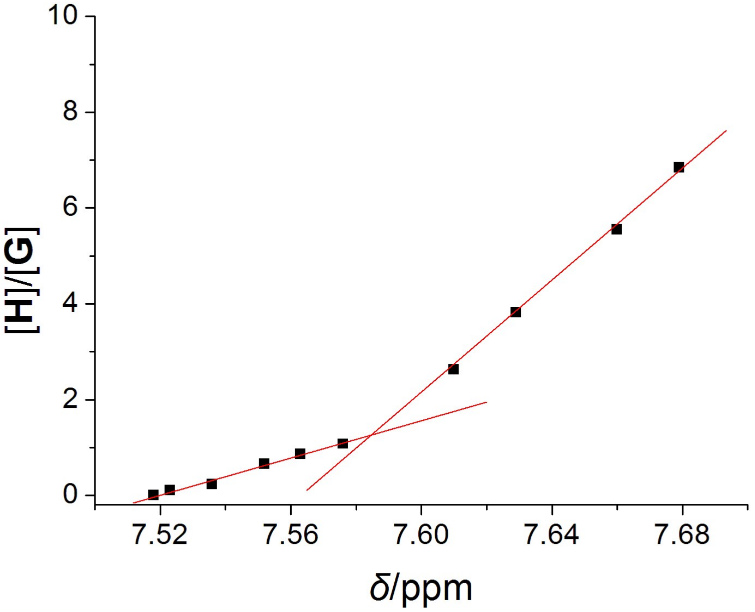

Fig. S33 Mole ratio plot for **P5** and **4**, indicating a 1:1 stoichiometry.

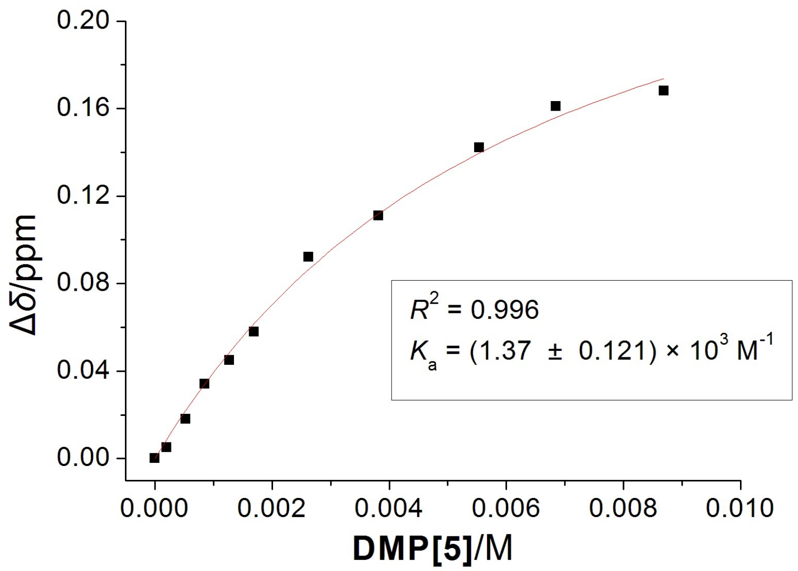

Fig. S34 The chemical shift changes of H_3 on **P5** upon addition of **4**. The red solid line was obtained from the non-linear curve-fitting using Eq. S1.

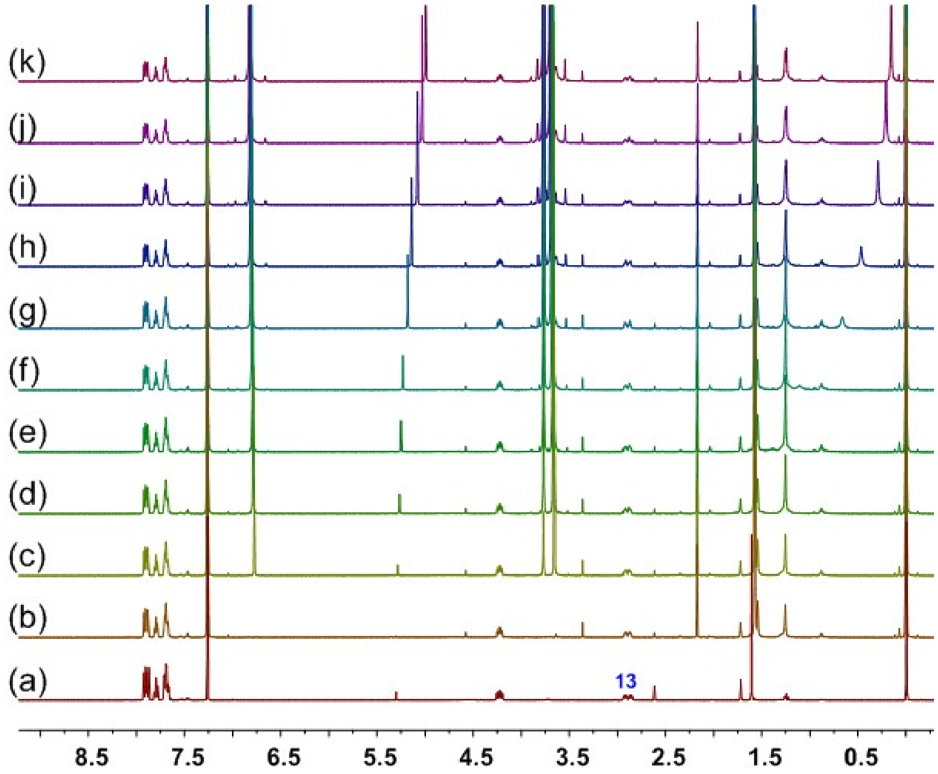

Fig. S35 Partial ^{1}H NMR spectra (500 MHz, chloroform-*d*, room temperature) of **5** at a concentration of 2.00 mM upon addition of **P5**: (a) 0 mM, (b) 0.100 mM, (c) 0.510 mM, (d) 1.16 mM, (e) 1.44 mM, (f) 1.98 mM, (g) 3.76 mM, (h) 5.36 mM, (i) 7.32 mM, (j) 9.42 mM, and (k) 12.6 mM.

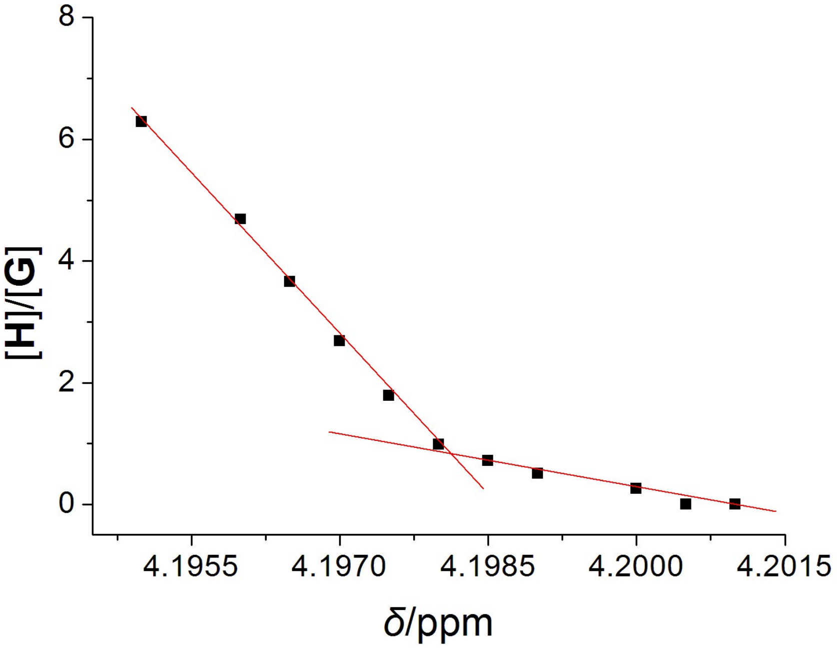

Fig. S36 Mole ratio plot for **P5** and **5**, indicating a 1:1 stoichiometry.

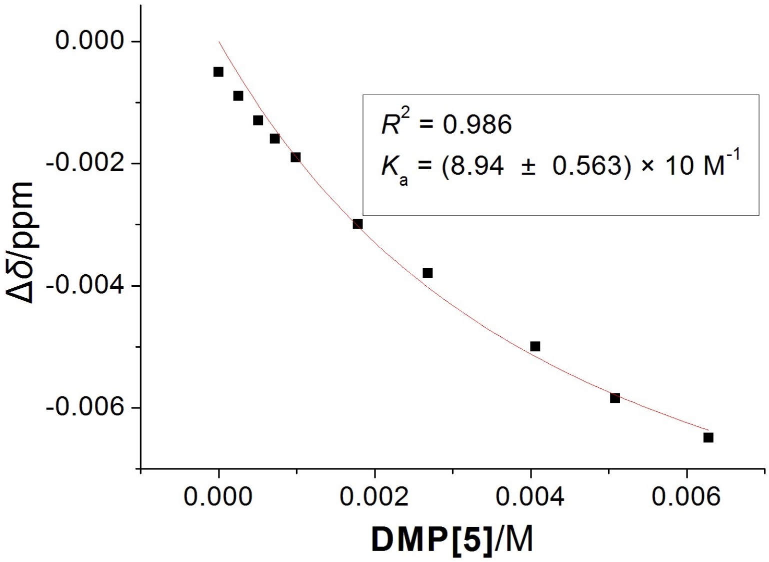

Fig. S37 The chemical shift changes of H_{13} on **P5** upon addition of **5**. The red solid line was obtained from the non-linear curve-fitting using Eq. S1.

^{1}H NMR titration experiments were carried out to determine the association constants and the complexation stoichiometries of the host–guest complexes. Mole ratio plots indicated that the stoichiometries for the host−guest complexes (**P5⊃4** and **P5⊃5**) were both 1:1 (Fig. S33 and Fig. S36). Meanwhile, the association constant (K_a) of **P5⊃4**

in chloroform-*d* was determined to be (1.37 ± 0.12) × 10^3 M^{-1} by a non-linear curve-fitting method, which was much higher than that 89.4 ± 5.6 M^{-1} of **P5⊃5** (Fig. S34 and Fig. S37).

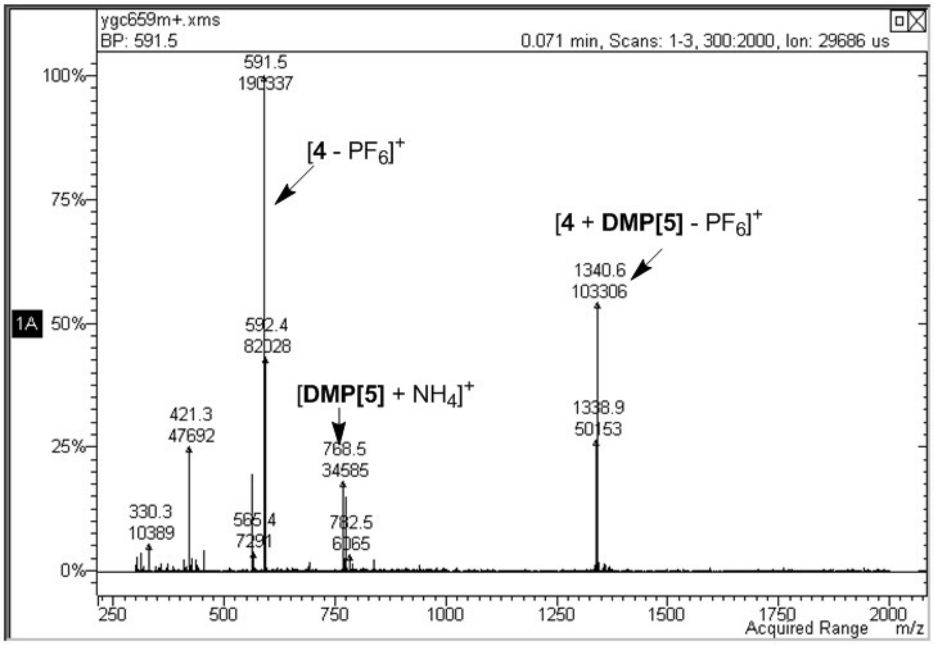

Fig. S38 Electrospray ionization mass spectrum of **P5⊃4**.

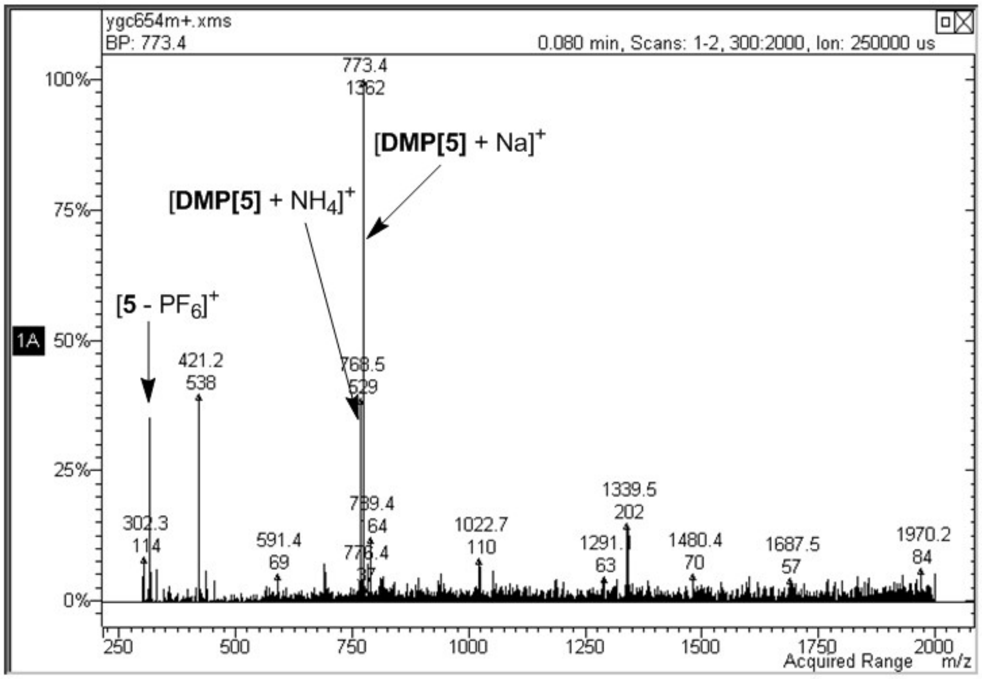

Fig. S39 Electrospray ionization mass spectrum of **P5⊃5**.

Further evidence for the formation of host–guest complex between **P5** and **4** was obtained from electrospray ionization mass spectrometry (ESI-MS). The relevant peak of the inclusion complex **P5⊃4** was found at *m*/*z* 1340.6, corresponding to [**P5⊃4** − PF6]$^{+}$, which confirmed the 1:1 complexation between **P5** and **4** (Fig. S38). However, other than the peaks at *m*/*z* 314.6, 768.5 and 773.4 corresponding to [**5** − PF_6]$^{+}$, [**P5** + NH_4]$^{+}$ and [**P5** + Na]$^{+}$, respectively, no peaks were observed related to the host–guest complex **P5⊃5** (Fig. S39), in good agreement with the results obtained from ^{1}H NMR investigations.

*4. Solvent-dependent molecular motion in **R1***

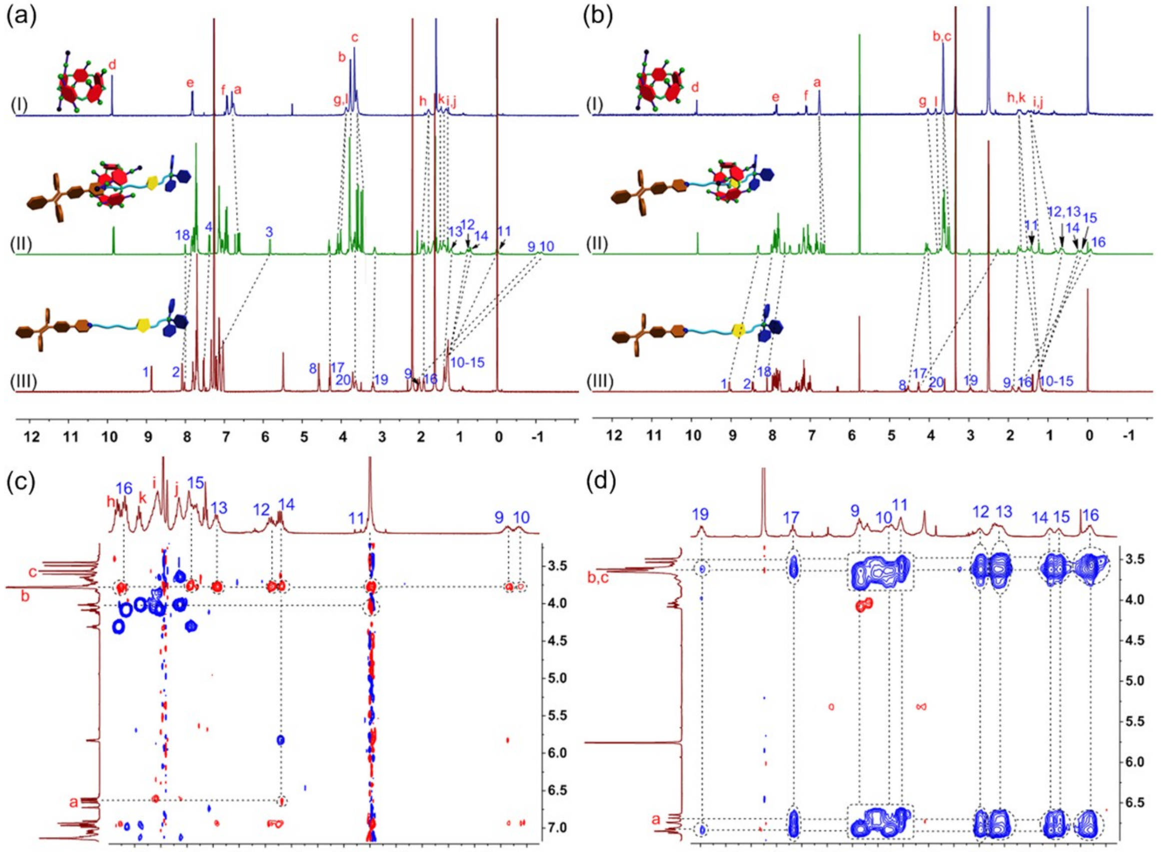

Fig. S40 ^{1}H NMR spectra (400 MHz, 295 K) in (a) chloroform-*d* and (b) DMSO-d_6: (I) **6** (2.00 mM), (II) **R1** (2.00 mM), (III) dumbbell-shaped component **7** (2.00 mM). Partial NOESY NMR spectrum of **R1**: (c) in chloroform-*d*, (d) in DMSO-d_6.

The ESI-MS data of **R1** contained a peak at *m*/*z* 1019.5 (Fig. S20), corresponding to [**R1** − $2PF_6$]$^{2+}$, which provided direct evidence for the formation of **R1**. From previous work, we knew that the pillar-shaped cavity moved along the long alkylene chain,

causing upfield shifts of the methylene protons. The pillar[5]arene ring was statistically located on the methylenes whose protons showed relatively bigger upfield shifts in solution. Therefore, we used ^{1}H NMR spectroscopy to determine the position of **6** in this MIM. On account of the relative high binding affinity between **P5** and **4**, we speculated that the cationic pyridinium ring and the adjacent methylene groups were located in the cavity of the pillar[5]arene ring. Indeed, the ^{1}H NMR and 2D NOESY spectra of **R1** in chloroform-*d* confirmed our deduction (Fig. S40, a and c). As shown in Fig. S40a, we found that the signals of pyridinium protons H_2, methylene protons $H_{9–12}$ and phenyl protons H_3 shifted upfield due to the shielding effect. For example, the signal of protons H_3 shifted upfield from 7.22 ppm to 5.84 ppm and the peaks of protons H_9 and H_{10} had chemical shifts even below 0 ppm in accordance with what happened for previously reported pillar[5]arene-based threaded structures with alkyl chain axles. Moreover, the signals corresponding to protons H^1 and H^8 disappeared upon formation of **R1** due to the extensive broadening effect. It should be noted that the resonances related to protons H_3, H_9, H_{10} and H_{11} of the axle showed much larger upfield chemical shift changes than other protons (Fig. S41), while for the peaks related to the protons $H_{17–20}$ near the TPP group, almost no chemical shift changes were observed. These phenomena meant that the cationic pyridinium ring and adjacent methylene groups were included in the cavity of the pillar[5]arene ring. The NOESY spectrum of **R1** in chloroform-*d* showed correlations between protons H_a, H_b, and H_c of the wheel and methylene protons $H_{9–16}$ of the axle, demonstrating the formation of the host–guest inclusion complex (Fig. S40c). In order to further verify this mechanically interlocked structure, the highly polar solvent DMSO-d_6 was used for proton NMR investigations. As shown in Fig. S40b, the peaks related to protons H_1, H_2, H_8, H_9 and $H_{12–18}$ displayed upfield chemical shifts due to the shielding effect, indicating that the axle was in the cavity of **4**. NOE correlation signals were observed between protons $H_{a–c}$ of the pillar[5]arene ring and protons $H_{9–17}$ and H_{19} on the axle in the NOESY spectrum of **R1** in DMSO-d_6 (Fig. S40d), convincingly confirming the formation of a MIM.

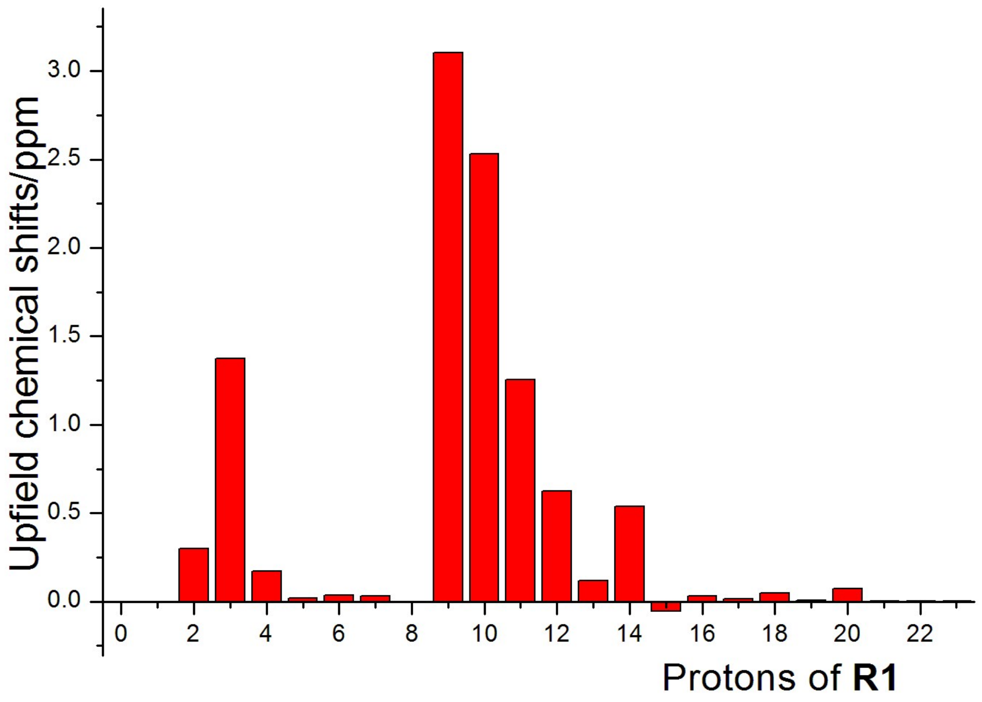

Fig. S41 Upfield chemical shifts of the protons on **R1** caused by the self-complexation in chloroform-*d*.

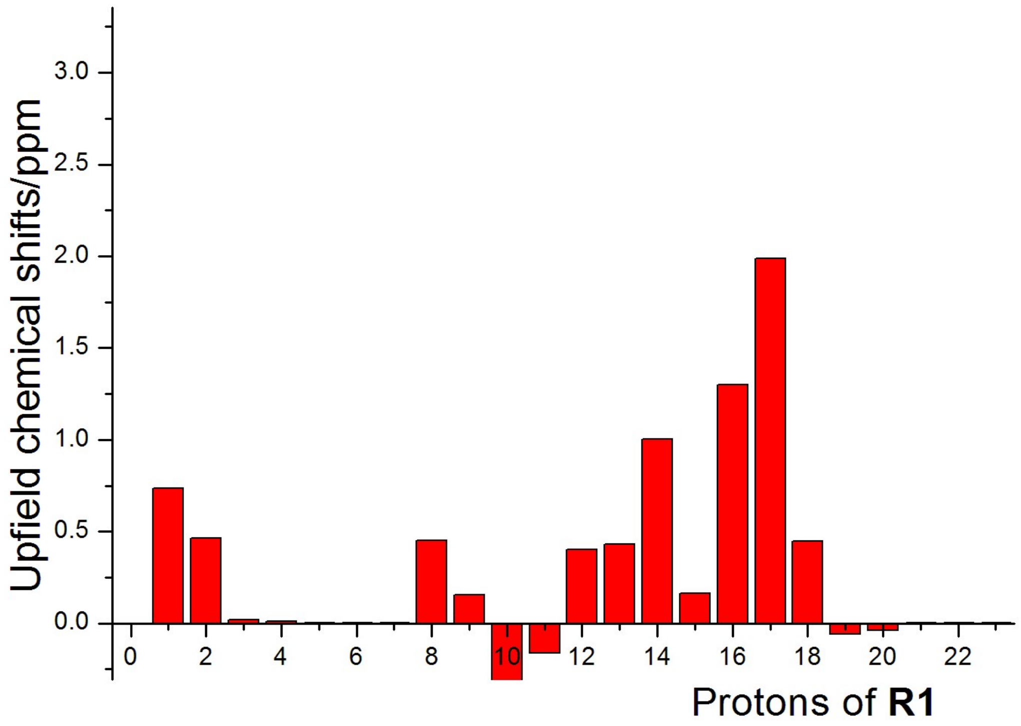

Fig. S42 Upfield chemical shifts of the protons on **R1** caused by the self-complexation in DMSO-d_6.

5. AIE property of 7

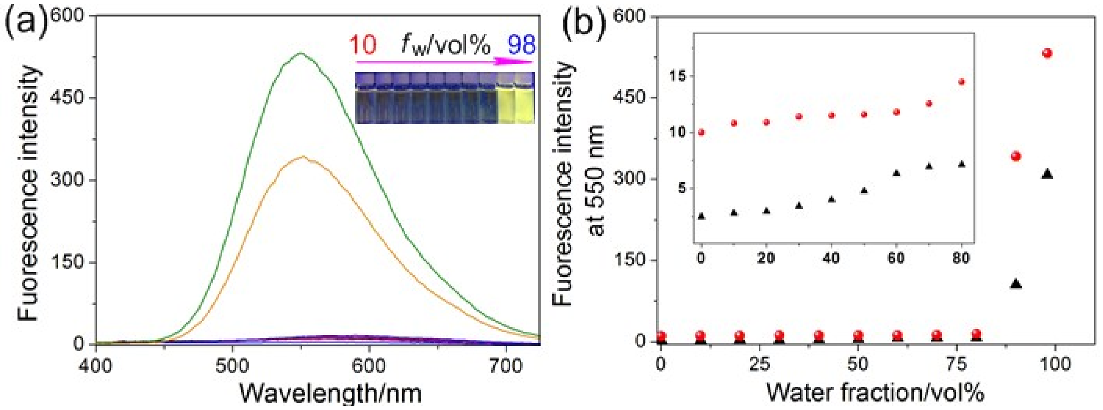

Fig. S43 (a) Fluorescence spectra of **R1** in mixtures of THF and water with different f_w values. Inset: a fluorescent photo of **R1** in mixtures of THF and water with different f_w values. (b) Plot of the emission intensity at 550 nm *vs* f_w of the aqueous mixtures: (▲) **7**; (●) **R1**. The concentrations of **7** and **R1** were 2.00×10^{-5} M.

The free axle **7** exhibited the characteristic AIE feature. It gave very weak emission in THF where it was well dissolved. The fluorescence intensity of **7** at 550 nm increased slowly with increasing volume fraction of water (f_w) in the THF/H_2O mixture from 0 to 80 vol%, and increased dramatically upon further enhancement of the f_w value from 80 to 98 vol% (Fig. S43), consistent with other classical AIE dyes.[13] Upon formation of a MIM, **R1** exhibited an enhancement of the AIE effect. It had faint fluorescence intensity when molecularly dissolved in THF, but was fluoresced intensively when the f_w value increased (Fig. S43a). It should be noted that the fluorescence intensity of **R1** was higher than that of **7** at the same concentration (Fig. S43b).

*6. Cytotoxicity evaluation of **R1** towards HeLa and HEK293 cells*

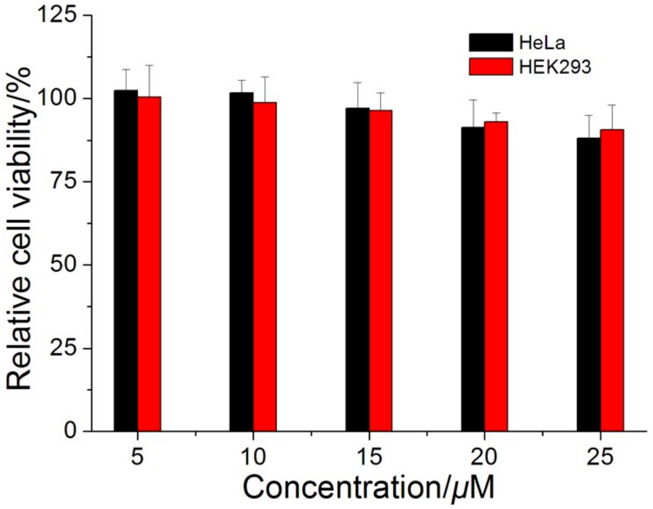

Fig. S44 Cytotoxicity of **R1** towards HeLa and HEK293 cells determined by MTT assays. The cells were incubated with **R1** NPs at different concentrations for 24 h.

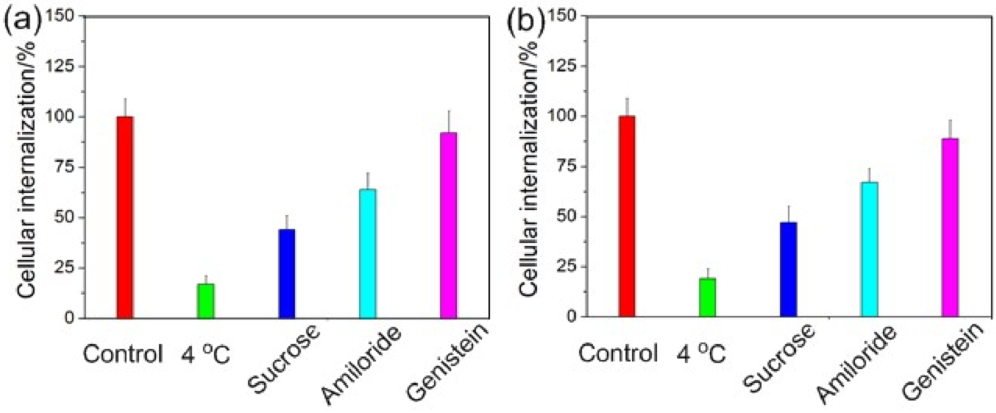

Fig. S45 Probing the mechanisms of cellular internalization by using various inhibitors, including sucrose, amiloride-HCl, and genistein. Percent internalization was normalized to **R1** NPs internalization in the absence of inhibitors.

NPs can enter cells by several different endocytic pathways, such as phagocytosis and pinocytosis, which not only affect the uptake efficiency of NPs but also their intracellular fate, affecting the pharmacological activities of the loaded cargoes (such as dyes, drugs or genes). Phagocytosis is conducted primarily by specialized cells, such as monocytes, macrophages and neutrophils, which can clear out large particles (several micrometers) in blood. Clathrin-mediated, macropinocytosis, caveolin-mediated, and clathrin- and caveolin-independent endocytosis are the four major processes of

pinocytosis, which operate in all mammalian cells. The internalization pathways of the NPs self-assembled from **R1** in HeLa and HEK293 cells were studied using flow cytometry by applying various endocytosis inhibitors (Fig. S45). The internalization pathways of the NPs self-assembled from **R1** were studied using flow cytometry by applying various endocytosis inhibitors. Uptake of **R1** NPs by the HeLa and HEK293 cells was almost completely inhibited at 4 °C (the low temperature is beneficial to minimize the metabolism of cell plasma membrane), demonstrating the energy-dependent nature of particle uptake (Fig. S45). HeLa cells treatment with sucrose resulted in a 56% decrease in the cellular uptake of **R1** NPs (Fig. S45a), suggesting that **R1** NPs might be mainly internalized *via* clathrin-mediated endocytic pathway, which generally plays an important role in the internalization of nanocontainers into cells. Additionally, a 36% decrease in the cellular uptake of **R1** NPs was monitored by treating HeLa cells with amiloride-HCl, indicating that the macropinocytosis-mediated pathway also partly contributed to the internalization of **R1** NPs. However, genistein, an inhibitor of caveolae-mediated endocytosis, did not show an obvious effect on the cellular uptake of **R1** NPs, demonstrating that the caveolae-mediated endocytosis pathway exerted a negligible effect on the uptake of **R1** NPs by the HeLa cells. Similarly, the cellular uptake of **R1** NPs by HEK293 cells was effectively blocked by sucrose and amiloride-HCl, suggesting that the internalization of these particles was mainly mediated by macropinocytosis- and clathrin-mediated endocytosis rather than the caveolae-mediated pathway (Fig. S45b). These pathways allowed **R1** NPs to undergo the endo/lysosomal transport for intracellular delivery of **R1**.

7. Photostability investigation of **R1** and MitoTracker Red in HeLa cells

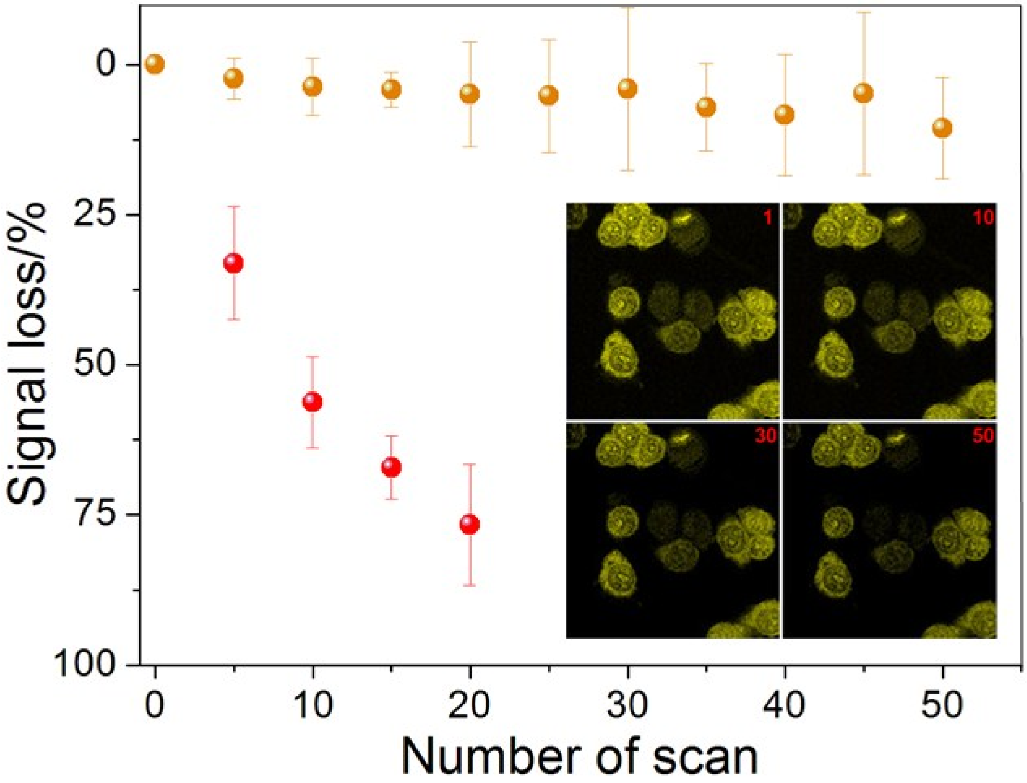

Fig. S46 Signal loss (%) of fluorescent emission of **R1** (●) and MitoTracker Red (●) with increasing number of scans. Inset: CLSM images of living HeLa cells stained with **R1** (2 μM) with increasing number of scans (the number of scans shown in upper right corner).

8. Förster resonance energy transfer in **R2**

According to the Förster theory, the transfer efficiency (Φ_T) can be expressed as:

$\Phi_T = k_T/(1/\tau_D + k_T) = 1 - \tau_{DA}/\tau_D$

Where τ_{DA} and τ_D are the fluorescence lifetime of the donor in the presence and absence of the acceptor, respectively. k_T is the transfer rate between an excited donor and an acceptor fluorophore.

$k_T = 1/\tau_D(R_0/r)^6$

Where r is the distance between the fluorophores.

The Förster radius (R_0, in Ångstrom) can be calculated using:

$R_0^6 = 8{:}79*10^{-5}\, k^2\, \Phi_D^0\, n^{-4}\, J(\lambda)$

Where Φ_D^0 is the fluorescence quantum yield of the donor in the absence of the

acceptor, n is the refractive index of the medium, $J(\lambda)$ is the overlap integral describing the degree of overlap between the donor fluorescence emission spectrum and the acceptor absorption spectrum, and k^2 is the orientation factor, a measure of the relative orientation of the transition dipole moments of the donor (emission) and the acceptor (absorption) and the vector connecting the molecules.

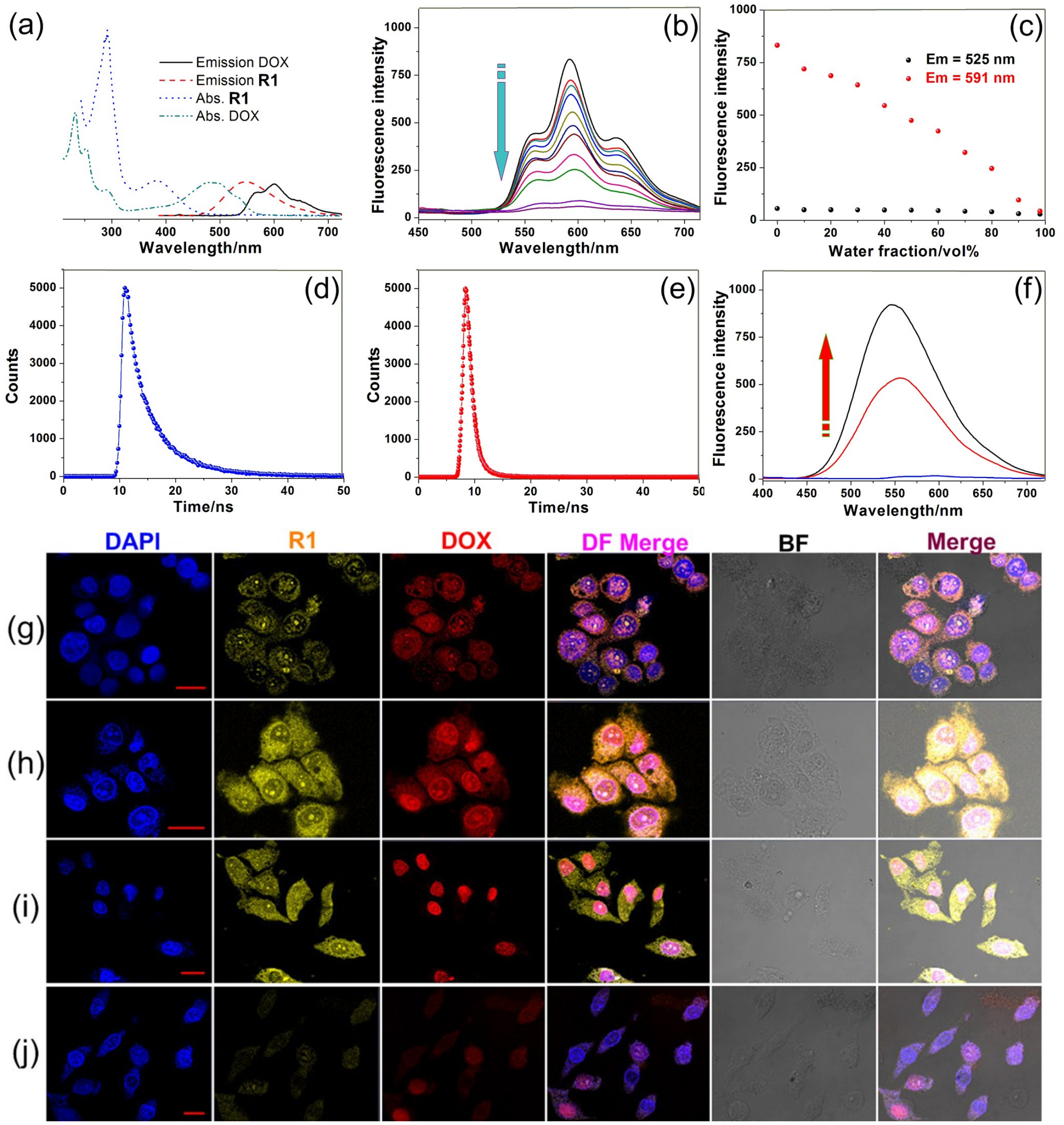

Fig. S47 (a) Absorption and fluorescence emission spectra of **R1** and DOX·HCl. (b) Fluorescence spectra of **R2** in THF/water mixtures with different f_w values. (c) Plot of emission intensities at 525 nm and 591 nm *vs* f_w of the aqueous mixture. Fluorescence lifetimes of (d) **R1**

and (e) **R2**. (f) Recovery of **R1** fluorescence by treating **R2** NPs with normal saline at pH 5.0 for 24 h (the free DOX was removed by dialysis). CLSM images depicting the subcellular distributions of **R1** and DOX in the (g−i) HeLa cells and (j) HEK293 cells. The incubation time was (g) 2 h, (h) 4 h, (i) 8 h, and (j) 8 h. Scale bar for all images = 20 μm.

Fig. S47a shows the absorption and fluorescence emission spectra of **R1** and doxorubicin hydrochloride (DOX·HCl), a typical anticancer drug which can be used for tracing drug delivery and cancer treatment. **R1** is excited by absorbing light with a wavelength of 400 nm, resulting in emission of light in the range of 450–700 nm. We found an overlap between the emission spectrum of **R1** and the absorption spectrum of DOX·HCl, confirming that **R1** could act as a fluorescent donor for the acceptor (DOX·HCl) that absorbs maximally at 500 nm. By the reaction between DOX·HCl and **R1**, DOX was grafted on **R1** by imine bonds formed between the amine group on the DOX and aldehyde groups on **R1**. As shown in Fig. S48, the NMR signals at 9.84 ppm of the aldehyde groups disappeared upon formation of **R2**, indicating that these groups were converted completely. Similary, NPs self-assembled from **R2** in water with an average diameter of about 60 nm were obtained through a reprecipitation technique (Fig. S49).

Next, we examined the ETR-caused dual-fluorescence quench behavior in **R2**. As shown in Fig. S47b, the characteristic emission corresponding to the TPE-based fluorogen was not observed for **R2**, indicating that the AIE behavior disappeared by introducing DOX into this MIM. The disappearance of the AIE behavior for **R2** was ascribed to the emissive energy transfer from the TPE-based fluorogen to DOX, because the distance between the donor and the acceptor was so short that FRET easily took place in **R2**. However, the ACQ effect of DOX in the aggregated state reduced the fluorescence intensity by "π-π stacking" of their rigid planar aromatic rings. To verify that the ACQ behavior occurred in **R2** NPs, a solvent dependent aggregation method was employed. The DOX chromophore in **R2** exhibited strong fluorescence at 591 nm when **R2** was well dissolved in THF. However, the fluorescence intensity dramatically decreased upon addition of water (Fig. S47, b and c). When the water fraction reached 98 vol %, the fluorescence intensity intensity of the DOX chromophore was nearly 19.3-fold weaker than that in pure THF (Fig. S47c), confirming that the ACQ effect of the DOX chromophore gradually increased along with the aggregation of **R2**. Thus, a dual-

fluorescence-quenched supramolecular system was prepared through ETR, in which the emission from **R1** was transferred to DOX, whereas the emission of DOX was self-quenched due to the ACQ effect.

Lifetime is a key kinetic parameter for the fluorescence intensity decay. Time-resolved spectroscopy was employed to investigate the photophysical behavior of **R1** and **R2** (Fig. 5, d and e). The decay dynamics of **R1** were better fitted by a double-exponential function, suggestting that two relaxation pathways were involved in the decay process. For example, 92.3% (A_1) and 7.7% (A_2) of the excitons of **R1** decayed *via* the fast and slow channels with lifetimes of 6.44 ns (τ_1) and 11.9 ns (τ_2), respectively. A possible explanation is that electron transfer to the pyridinium unit occurs in a reversible fashion.[S4] For **R2**, the excited state decayed in a three-exponential fashion with short components τ_1 = 1.01 ns (90.3%) and τ_2 = 2.54 ns (7.1%) that predominated at short wavelengths, and a longer decay time τ_3 = 9.9 ns (2.6%). A tentative explanation for this observation is that the noncovalent interactions between the TPE-based axle and the wheel led to an exciplex or heteroexcimer.[S4] The weighted mean lifetimes (τ) of **R1** and **R2** were calculated to be 6.86 ns and 1.29 ns, respectively. From these analyses, the efficiency of energy transfer (Φ_T) of this system was calculated to be 81%, and the Förster radius (R_0) and the energy transfer rate (k_T) were estimated as 1.99 nm and 0.77, respectively. In the present case, the value of Φ_T was quite high, indicating that a very efficient energy transfer took place from the TPE-based chromophore to the wheel unit. Moreover, the distance between the donor and the acceptor was calculated to be 1.56 nm, which is in good agreement with the molecular structure.

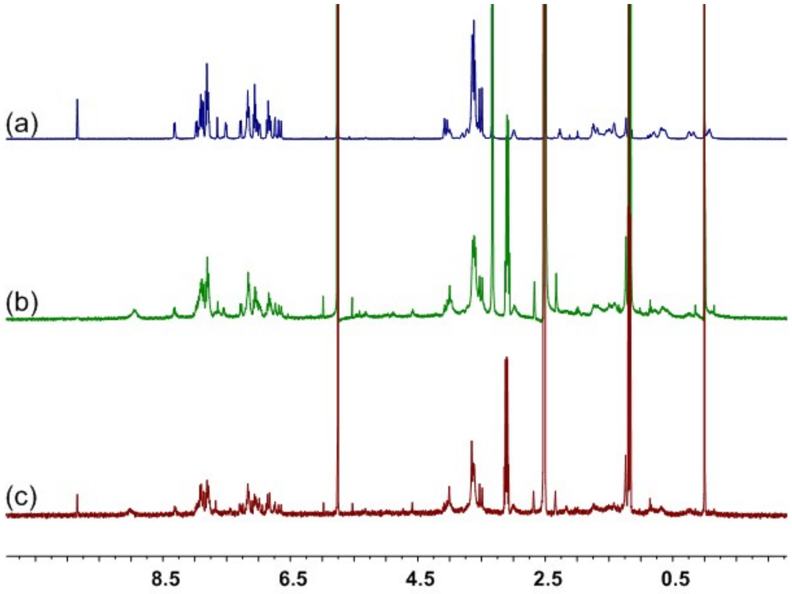

Fig. S48 ^{1}H NMR spectra (400 MHz, chloroform-*d*, room temperature): (a) **R1**; (b) **R2**; (c) **R2** in the presence of TFA.

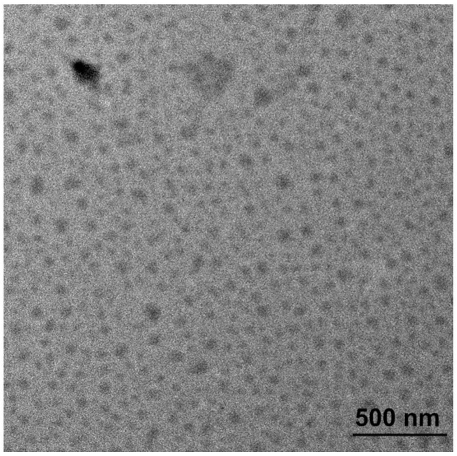

Fig. S49 TEM image of **R2** NPs in water.

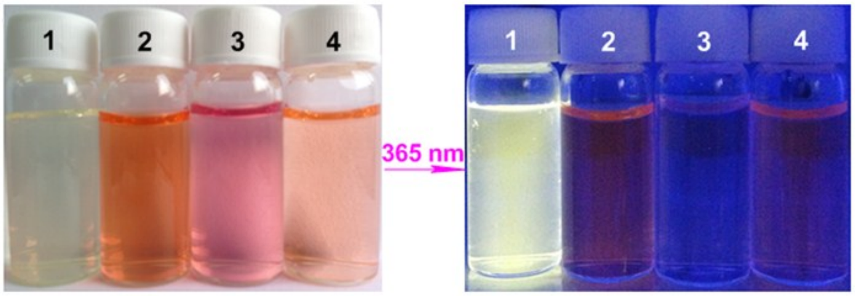

Fig. S50 Dual-color responsiveness of **R2** when it encounters the low pH circumstance. Pictures of (1) **R1**, (2) DOX, (3) **R2**, and (4) **R2** (pH 5.0, treated for 24 h) under laboratory lighting (left) and under a UV lamp with laser excitation at 365 nm (right).

The fluorescence recoveries of **R2** NPs at different pH values were visualized by using a UV lamp with an excitation wavelength of 365 nm. As indicated by fluorecent spectra shown in Fig. S50 and Fig. S51, the fluorescence intensity of DOX recovered effectively when the solution pH was adjusted to 5.0, demonstrating that DOX was released from **R2** NPs. Unexpectedly, no obvious fluorescence intensity recovery was observed when we monitored the fluorescence recovery of **R1** by culturing the **R2** NPs at pH 5.0. A possible reason is the persistence of FRET between the **R1** and DOX molecules. After dialysis, the detached DOX was separated from **R1**, and the fluorescence intensity corresponding to **R1** recovered significantly, indicating that the AIE behavior of **R1** was indeed retained. This phenomenon verified that **R1** remained in the aggregated state and retained its AIE behavior when DOX was released from the **R2** NPs. The fluorescence intensity of **R1** and DOX was recovered, affording a dual-color fluorogenic process once the imine bonds were cleaved.

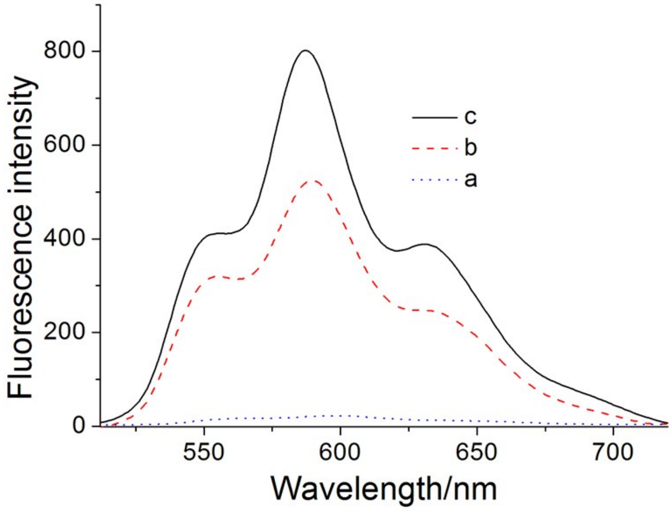

Fig. S51 Fluorescence spectra of (a) **R2**, (b) **R2** treated with normal saline at pH 5.0 for 24 h, and (c) free DOX·HCl.

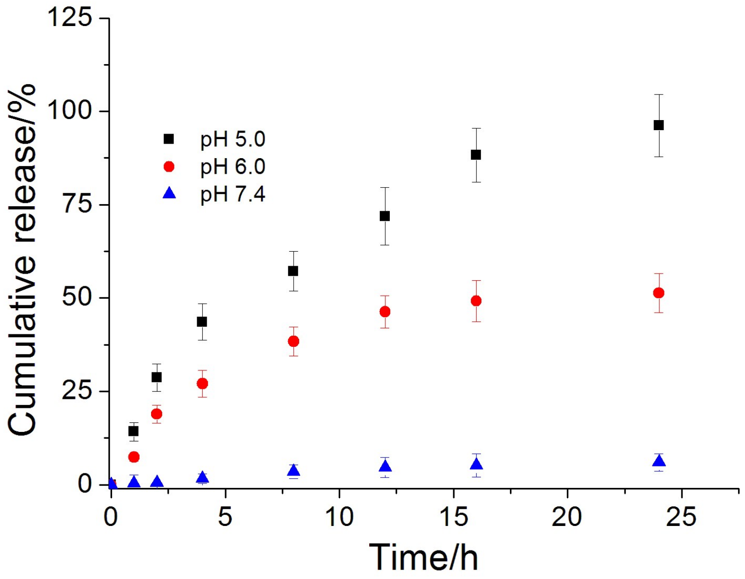

Fig. S52 Release profiles of DOX from **R2** NPs at different pH values.

The intracellular microenvironment of tumor cells is typically characterized by slightly acid pH in the endosomal (5.0−6.0) and lysosomal (4.0−5.0) compartments. When the pH was adjusted to 5.0 upon addition of DCl, the imine bonds were broken and **R2** decomposed into **R1** and free DOX. Actually, when the pH value was changed to 6.5, the signal at 9.84 ppm corresponding to the aldehyde group appeared again (Fig. S48), confiming the hydrolysis of **R2**. The release behavior of DOX from **R2** NPs was carried out at pH 7.4, 6.0, and 5.0, respectively, mimicking the pH gradient from blood circulation to the endo/lysosomal compartments. As shown in Fig. S52, about 6.0% of DOX was released within 24 h at pH 7.4. However, 51.3% of DOX was released from **R2** NPs after 24 h at pH 6.0 and nearly 100% at pH 5.0, respectively. The DOX release profile was clearly pH-dependent, owing to the accelerated hydrolysis of the imine bonds. The DOX release from **R2** NPs was switched off during systematic circulation (pH 7.4). However, prompt DOX release occurred upon being entrapped in the endo/lysosomal compartments after endocytosis.

9. Fabrication of nanoprodrugs

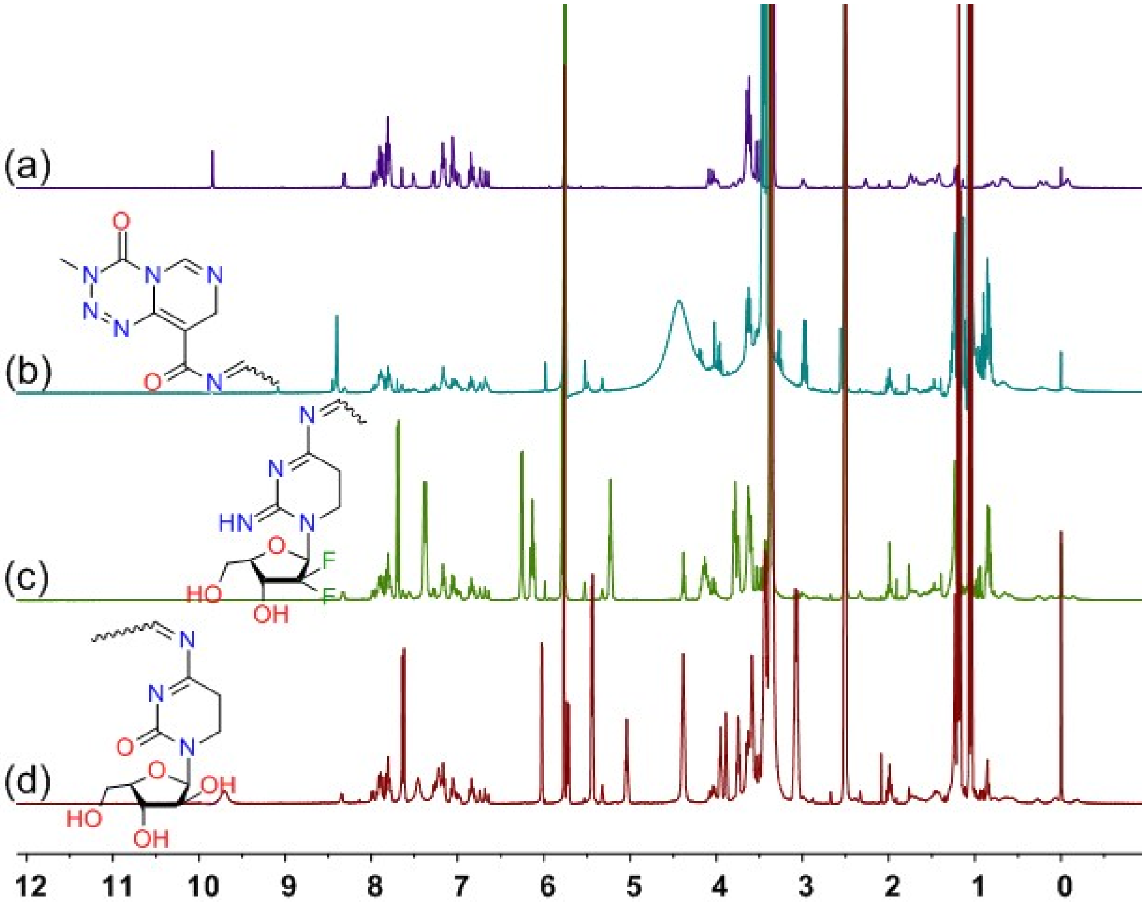

Fig. S53 ^{1}H NMR spectra (400 MHz, chloroform-*d*, room temperature): (a) **R1**; (b) **RTe**; (c) **RGe**, (d) **RCy**.

As shown in Fig. S53, the NMR signal at 9.84 ppm of the aldehyde groups disappeared upon formation of the prodrugs, indicating that these groups were converted completely.

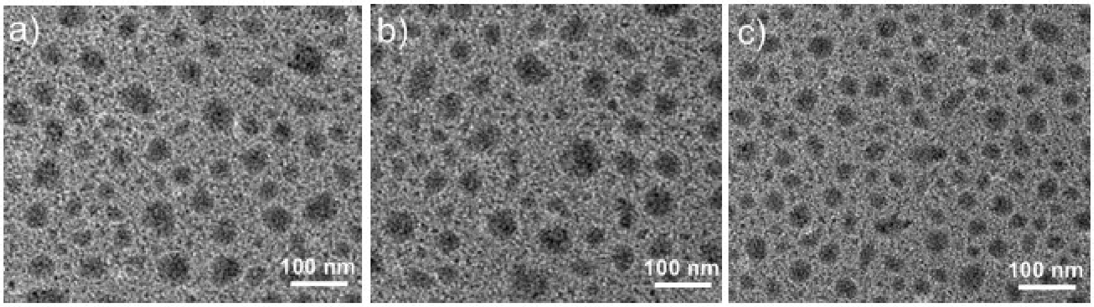

Fig. S54 TEM images of (a) **RTe** NPs, (b) **RGe** NPs, and (c) **RCy** NPs in water.

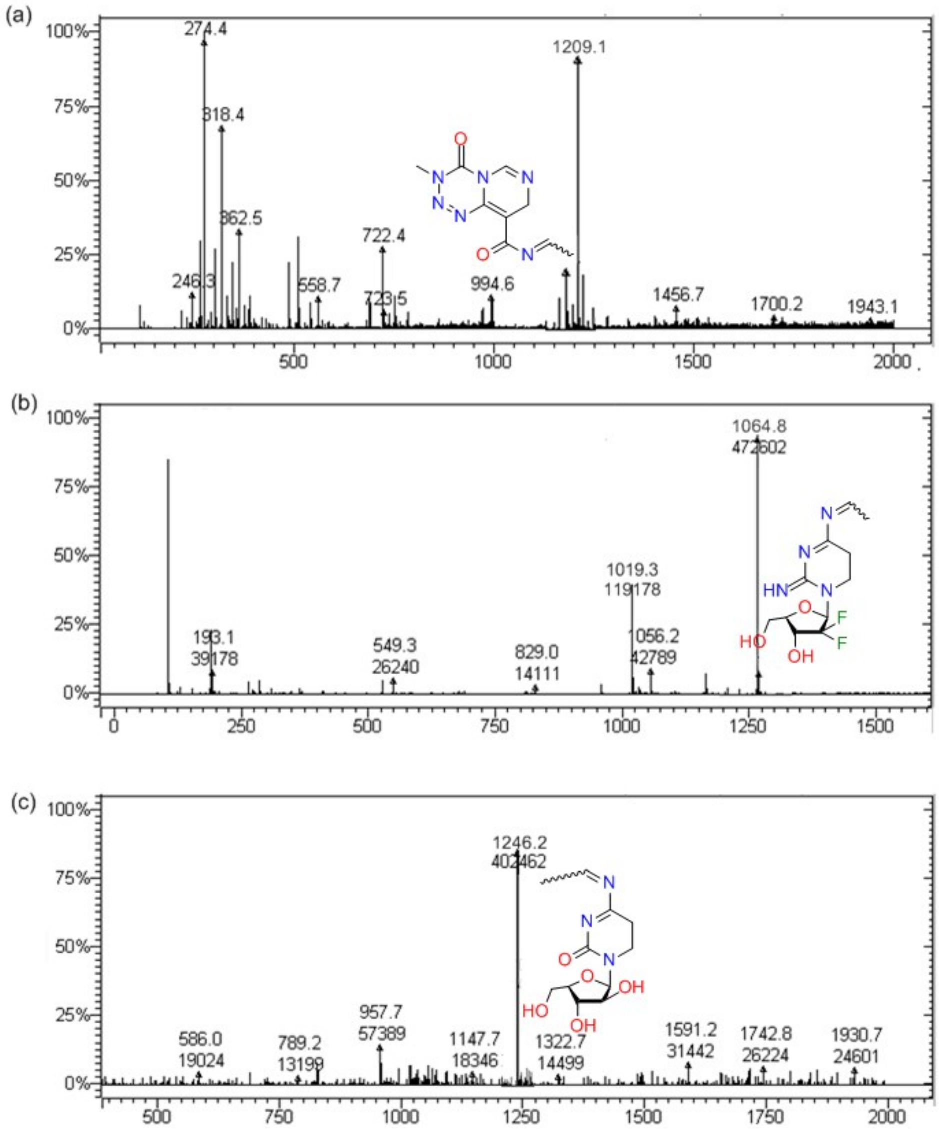

Fig. S55 Electrospray ionization mass spectrum of (a) **RTe**, (b) **RGe** and (c) **RCy**.

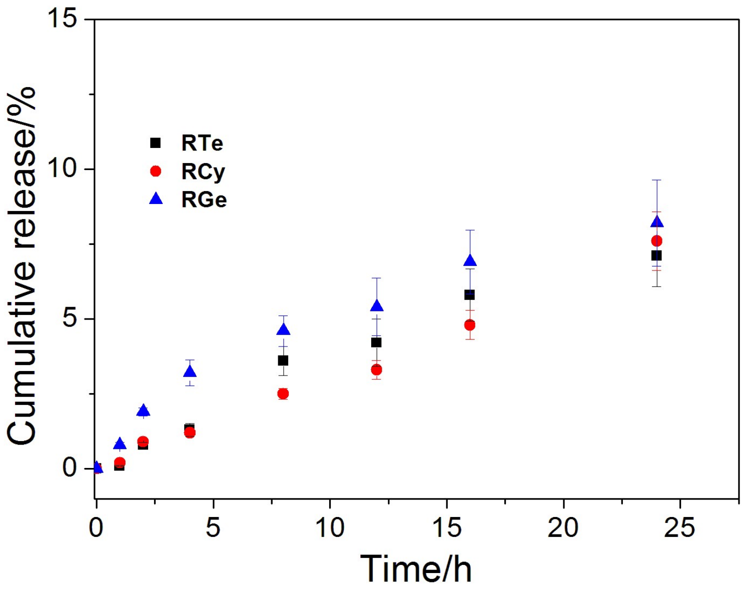

Fig. S56 Release profiles of the drugs from the prodrug NPs in PBS at pH 7.4 in the presence of 10% FBS.

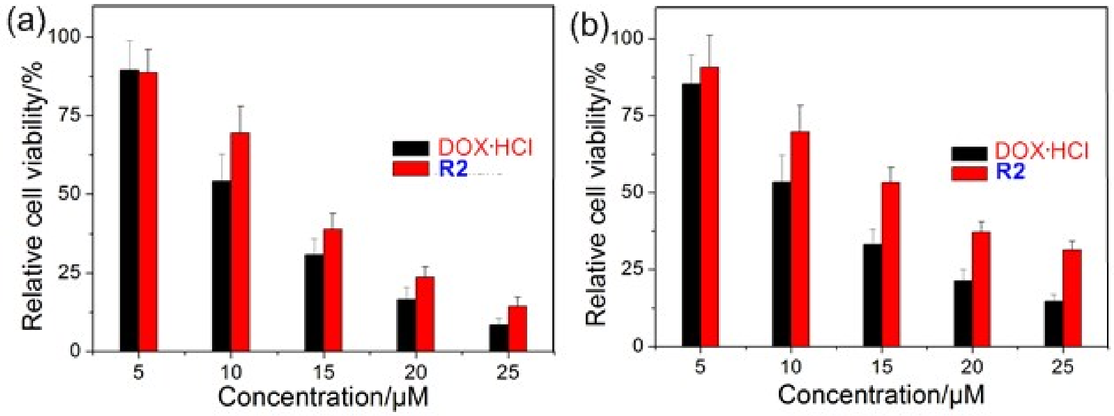

Fig. S57 Relative cell viabilities of (a) HeLa cells and (b) HEK293 cells incubated with **R2** NPs at different concentrations for 24 h.

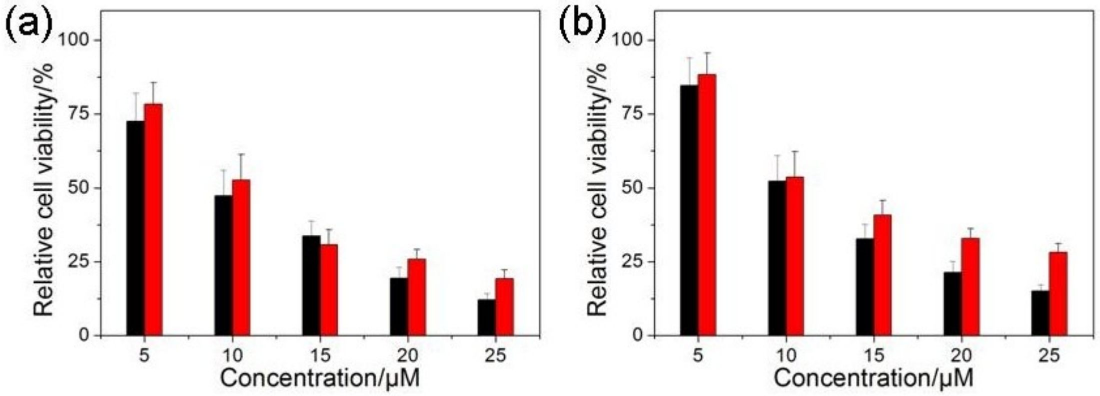

Fig. S58 Relative cell viabilities of (a) U-87-MG cells and (b) HEK293 cells incubated with **RTe** NPs (red column) and free temozolomide (black column) at different concentrations for 24 h.

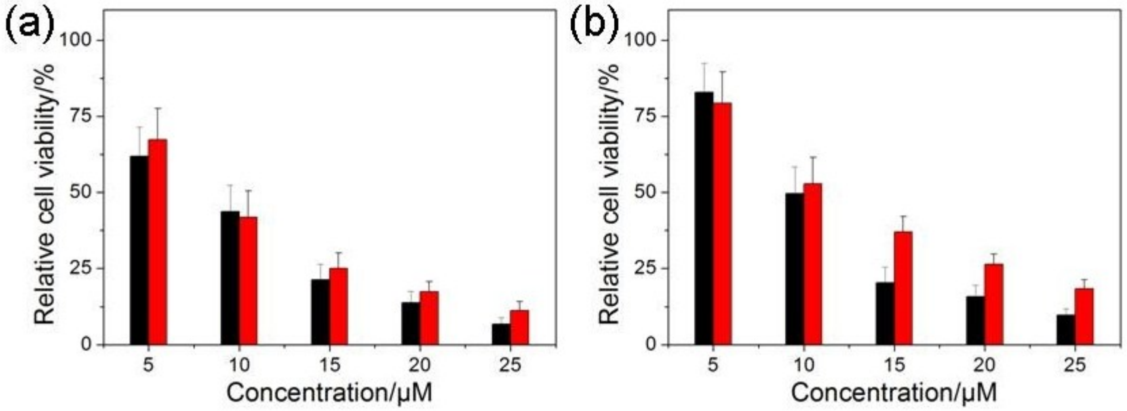

Fig. S59 Relative cell viabilities of A549 cells and (b) HEK293 cells incubated with **RGe** NPs (red column) and free gemcitabine (black column) at different concentrations for 24 h.

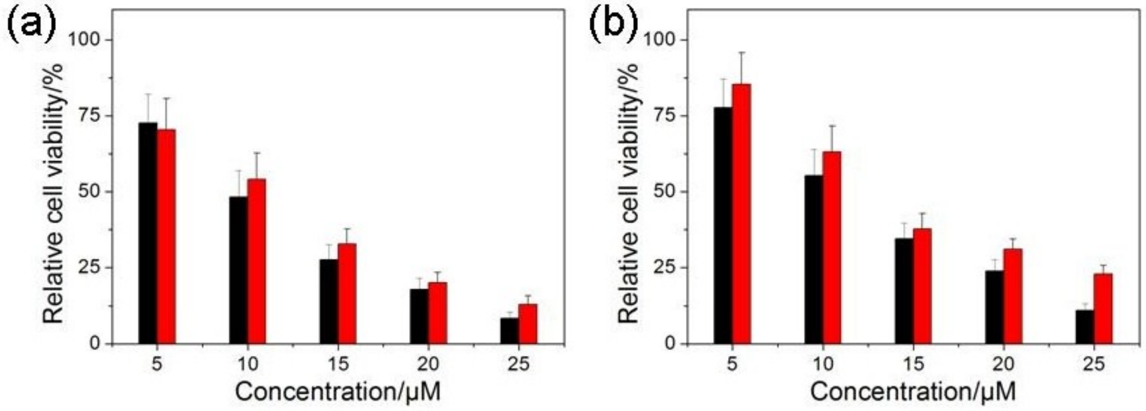

Fig. S60 Relative cell viabilities of L1210 cells and (b) HEK293 cells incubated with **RCy** NPs (red column) and free cytarabine (black column) at different concentrations for 24 h.

The results discussed in the main text indicated that DOX was released in endo/lysosomal compartments due to the hydrolysis of Schiff base linkages in mildly acidic environments. To assess the antitumor activity of the released DOX *in vitro*, we evaluated its cytotoxicity towards HeLa and HEK293 cell lines by using MTT assays. The cytotoxicity of each treatment was expressed as the percentage of cell viability relative to the untreated control cells. MTT assays were performed by exposing the cells to free DOX·HCl and **R2** NPs with concentrations ranging from 5 to 25 μM. As shown in Fig. S55a, the relative cell viability of the HeLa cells incubated with **R2** NPs decreased gradually from 88.7% to 14.3% upon increasing the concentration of **R2** NPs from 5 to 25 μM, confirming that the released DOX retained anticancer activity. It should be noted that the relative cell viability of the HeLa cells incubating with **R2** NPs was higher than the HeLa cells cultured with free DOX·HCl under the same conditions, because the cationic DOX·HCl with good water solubility diffused into the cells easily, so the concentration of the anticancer drug was higher than that released by the hydrolysis of **R2** NPs. On the other hand, the relative cell viability of the HeLa cells was lower than that of the HEK293 cells at the same concentration of **R2** NPs (Fig. S55b), because carcinoma cells have a higher membrane potential than normal cells, thus resulting in the improvement of the cellular uptake of **R2** NPs. Another reason for the difference in cytotoxicity of **R2** NPs towards HeLa and HEK293 cells was the difference in intracellular pH values. Typically, the intracellular pH in cancer cells is lower than that of normal cells. Therefore, the anticancer drug conjugated on the rotaxane was released faster in HeLa cells than in HEK293 cells.

References

S1 X.-F. Duan, J. Zeng, J.-W. Lü and Z.-B. Zhang, *J. Org. Chem*., 2006, **71**, 9873–9876.

S2 L. Liu, D. Cao, Y. Jin, H. Tao, Y. Kou and H. Meier, *Org. Biomol. Chem*., 2011, **9**, 7007–7010.

S3 K. A. Connors, *Binding Constants*, Wiley: New York, 1987. (b) P. S. Corbin, *Ph.D. Dissertation*, University of Illinois at Urbana-Champaign, Urbana, IL, 1999. (c) P. R. Ashton, R. Ballardini, V. Balzani, M. Belohradsky, M. T. Gandolfi, D. Philp, L. Prodi, F. M. Raymo, M. V. Reddington, N. Spencer, J. F. Stoddart, M. Venturi and D. J. Williams, *J. Am. Chem. Soc.*, 1996, **118**, 4931–4951. (d) J. Zhang, F. Huang, N. Li, H. Wang, H. W. Gibson, P. Gantzel and A. L. Rheingold, *J. Org. Chem.*, 2007, **72**, 8935–8938.

S4 T. Ogoshi, D. Yamafuji, T.-a. Yamagishi and A. M. Brouwer, *Chem. Commun*., 2013, **49**, 5468–5470.
